# Expression, Purification and Biophysical Characterisation of *Klebsiella Pneumoniae* Protein Adenylyltransferase: A Systematic Integration of Empirical and Computational Modelling Approaches

**DOI:** 10.1007/s10930-024-10210-3

**Published:** 2024-07-09

**Authors:** Reabetswe Maake, Ikechukwu Achilonu

**Affiliations:** Protein Structure‑Function and Research Unit, School of Molecular and Cell Biology, Faculty of Science, University of the Witwatersrand, Braamfontein, Johannesburg, 2050 South Africa

**Keywords:** *Klebsiella pneumoniae*, Protein adenylyltransferase, Recombinant protein expression, Biophysics, Isothermal titration calorimetry, Computational modelling

## Abstract

Infections that are acquired due to a prolonged hospital stay and manifest 2 days following the admission of a patient to a health-care institution can be classified as hospital-acquired infections. *Klebsiella pneumoniae* (*K. pneumoniae*) has become a critical pathogen, posing serious concern globally due to the rising incidences of hypervirulent and carbapenem-resistant strains. Glutaredoxin is a redox protein that protects cells from oxidative stress as it associates with glutathione to reduce mixed disulfides. Protein adenylyltransferase (PrAT) is a pseudokinase with a proposed mechanism of transferring an AMP group from ATP to glutaredoxin. Inducing oxidative stress to the bacterium by inhibiting the activity of PrAT is a promising approach to combating its contribution to hospital-acquired infections. Thus, this study aims to overexpress, purify, and analyse the effects of ATP and Mg^2+^ binding to *Klebsiella pneumoniae* PrAT (*Kp*PrAT). The pET expression system and nickel affinity chromatography were effective in expressing and purifying *Kp*PrAT. Far-UV CD spectroscopy demonstrates that the protein is predominantly α-helical, even in the presence of Mg^2+^. Extrinsic fluorescence spectroscopy with ANS indicates the presence of a hydrophobic pocket in the presence of ATP and Mg^2+^, while mant-ATP studies allude to the potential nucleotide binding ability of *Kp*PrAT. The presence of Mg^2+^ increases the thermostability of the protein. Isothermal titration calorimetry provides insight into the binding affinity and thermodynamic parameters associated with the binding of ATP to *Kp*PrAT, with or without Mg^2+^. Conclusively, the presence of Mg^2+^ induces a conformation in *Kp*PrAT that favours nucleotide binding.

**Supplementary Information:**

The online version contains supplementary material available at 10.1007/s10930-024-10210-3.

## Introduction

Nosocomial infections, or hospital-acquired infections (HAIs), arise within healthcare institutions after a patient’s admission and typically manifest around two days later [1]. Apart from their significant morbidity and mortality rates, HAIs also impose substantial financial burdens, costing around €7 billion in developed countries [2, 3]. The prevalence of HAIs vary globally, reaching up to 12% in developed nations, 15% in developing countries, and as high as 49% in sub-Saharan Africa [4]. Particularly vulnerable are patients in intensive care units (ICUs) and neonatal units, where HAIs account for 40% of neonatal deaths in developing countries and have a prevalence of over 20% in ICU patients [5, 6]. HAIs often spread through invasive medical devices like catheters and ventilators and can be categorised into types such as catheter-associated urinary tract infections (CAUTI), central line-associated bloodstream infections (CLABSI), and ventilator-associated pneumonia (VAP) [7, 8]. These infections are caused by multi-drug resistant (MDR) bacteria known as ESKAPE pathogens, including *Enterococcus faecium*, *Staphylococcus aureus*, *Klebsiella pneumoniae*, *Acinetobacter baumannii*, *Pseudomonas aeruginosa*, and *Enterobacter species* [9, 10]. Of particular concern is *Klebsiella pneumoniae* (*K. pneumoniae*), which is responsible for hospital-associated pneumonia and urinary tract infections (UTIs) [11]. It exhibits resistance to most β-lactam antibiotics and carbapenems, leading to the emergence of extended-spectrum β-lactamases (ESBL)-producing *K. pneumoniae* and carbapenem-resistant *K. pneumoniae* (CRKP) strains, partly due to the overuse of antibiotics in healthcare and agriculture [11, 12].

The current treatment strategies against MDR bacteria include antimicrobial peptides, antibiotic combination therapy, and bacteriophage therapy [13, 14]. Antimicrobial peptides are naturally or chemically synthesised compounds with antibacterial and anti-biofilm properties. They have been shown to be effective against *Staphylococcus aureus* [15]. Antibiotic combination therapy employs multiple antibiotics to treat infections. One way that combination therapy could prove to be effective would be to impede the same target in various ways [14]. However, this approach was ineffective for *Pseudomonas aeruginosa* because one antibiotic impeded the action of the second antibiotic [16]. Natural bacterial viruses and bacteriophages infect bacteria and can potentially be therapeutic agents against MDR bacteria [17]. They are advantageous as they have high specificity for the bacterial pathogen, with little to no effect on humans [18]. The novel therapeutic approach targeted against MDR *K. pneumoniae* and one that will be explored in this study would be to target the reduction-oxidation (redox) homeostasis of the bacterium through the pseudokinase protein adenylyltransferase (PrAT).

The emergence of photosynthesis has increased atmospheric oxygen [19]. This has led to biological processes dependent on oxygen, such as aerobic respiration, and consequently, driving the need for mechanisms that modulate the balance between reduction and oxidation reactions. Redox reactions are pivotal in cellular regulation processes such as cell death, development, differentiation, and signalling [20]. Reactive oxygen species (ROS) are an outcome of aerobic respiration, and they are oxidants. These species inhibit the activity of antioxidant proteins. Furthermore, they oxidise cysteine residues in proteins, thus rendering the protein inactive [21, 22]. ROS contributes to redox imbalance through its accumulation. This imbalance leads to oxidative stress in cells and causes cell damage [23]. Antioxidant species such as glutathione are employed to counter ROS’s influence. Glutathione prevents the oxidation of cysteine residues [24].

**Fig. 1:**
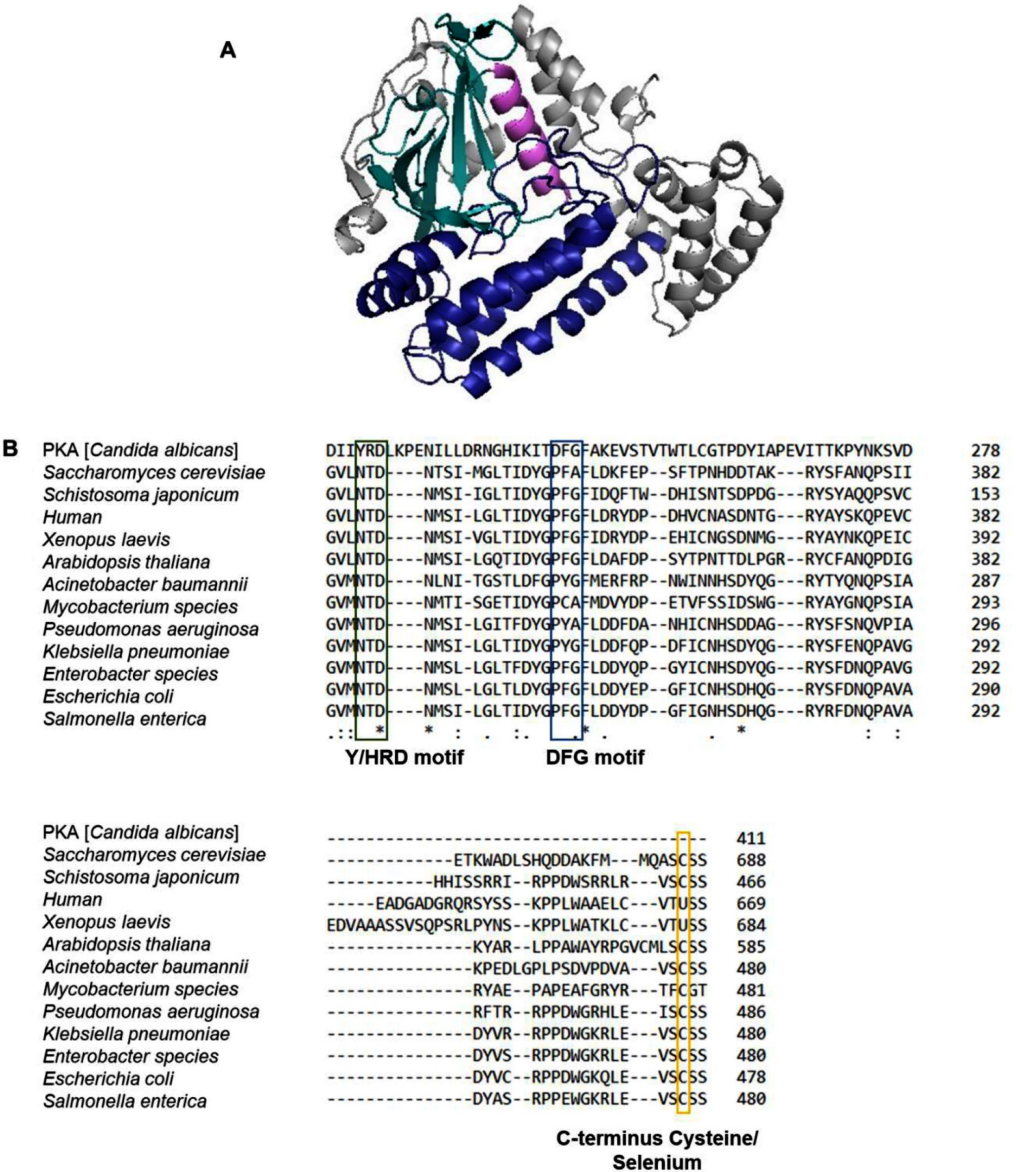
Homology model and multiple sequence alignment of *Kp*PrAT. (**A**) Ribbon structure of the homology model of *Kp*PrAT (PDB:6K20). The β-sheet found in the N-lobe is represented as a deep teal, while the αC-helix is a violet purple. The C-lobe is displayed as a deep blue. (**B**) Multiple sequence alignment shows the conserved positions in SelO proteins and *Candida albicans* protein kinase A (PKA [*Candida albicans*]). The Y/HRD motif is missing for SelO proteins relative to PKA [*C. albicans*] and enclosed in a green square. Furthermore, the aspartate in the DFG motif is missing for all SelO proteins, represented by blue square. However, there is a DYG motif that precedes the mutated DFG motif across most species. Most eukaryotic SelO proteins contain a Sec at their C-terminus, but bacterial SelO *proteins* have a Cys, represented by a yellow square

Protein kinase transfers a γ-phosphate group from ATP to a hydroxyl-containing amino acid such as threonine, tyrosine, and serine in a substrate protein. This group of enzymes is involved in a plethora of biological processes such as cell signalling and regulation [25]. Protein kinases consist of two lobes, N-terminus, and C-terminus. The N-lobe has five β-strands (β_1_-β_5_) that form a β-sheet, followed by a single α-helix, αC-helix. The C-lobe is predominantly α-helical and has a 2-stranded β-sheet (β_6_-β_7_). Within the C-lobe is an aspartate-phenylalanine-glycine (DFG) motif, the aspartate residue fronting towards the ATP-binding site, further aiding in the binding of magnesium to ATP. The tyrosine/histidine-arginine-aspartate (Y/HRD) motif forms part of the catalytic loop and stabilises the active conformation of the protein kinase [26]. A group of kinases that are considered inactive because they lack residues essential in protein kinase activity are referred to as pseudokinases [27]. These enzymes are allosteric regulators of kinases. The pseudokinase, selenoprotein-O (SelO) or protein adenylyltransferase (PrAT), catalyses the transfer of an α-phosphate group from ATP to a serine, threonine, or tyrosine residue protein. It has the conventional structure of a protein kinase but lacks the aspartate in the DFG motif, essential in protein kinase catalysis and the Y/HRD motif (Figure 1). Furthermore, it lacks the C-terminus selenocysteine (Sec) found in most SelO proteins [28]. SelO proteins are Sec-containing proteins. Sec is an amino acid that is structurally like cysteine but has selenium as opposed to sulfur. The pK_a_ of the selenium is low; thus, at physiological pH, the amino acid is deprotonated, which leads to a high nucleophile capacity and is highly reactive [29]. PrAT has been shown to AMPylate glutaredoxin (Grx), involved in redox homeostasis [28]. Through this association, PrAT indicates its potential as an appropriate drug target for achieving oxidative stress in bacterial species. This research aims to recombinantly over-express, biophysically characterise *Klebsiella pneumoniae* protein adenylyltransferase (*Kp*PrAT) using a systematic integration of empirical and computational modelling studies. Outcomes from this study would be critical in understanding the endogenously and exogenous molecular targets of this enzyme as well designing crystallisation conditions for determination of the three-dimensional structure of the enzyme.

## Materials and Methods

### Materials

Unless otherwise specified, all reagents were of analytical grade and obtained from Sigma-Aldrich (St. Louis, MO, USA). The expression vector pET-11a-*Kp*PrAT was constructed by GenScript (Piscataway, NJ, USA).

### Construction of the 6His-*Kp*PrAT Vector

The cDNA gene encoding *Kp*PrAT (UniProtKB accession B5XQE2) was cloned into a pET-11a expression vector. The nucleotide sequence of *Kp*PrAT was inserted between the *Nde* I (5ʹ-CATATG-3ʹ) and *BamH* I (5ʹ-GGATCC-3ʹ) restriction sites, with the gene encoding an N-terminal His-tag. The nucleotide sequence was synthesised by GenScript (NJ, USA) and cloned into a pET-11a vector. The resulting pET-11a-*Kp*PrAT vector construct is illustrated in Figure S1A. DNA sequencing was performed by Inqaba Biotec (Pretoria, RSA).

### Overexpression of Recombinant *Kp*PrAT

The *E. coli* T7 cells from New England BioLabs (Pretoria, RSA) were rendered competent and then subjected to transformation with the pET-11a-*Kp*PrAT construct. Following this transformation, *E. coli* overnight cultures were freshly prepared by introducing single colonies from LB-agar into 2×YT media, enhanced with 100 µg·mL^− 1^ ampicillin. Incubation of these overnight cultures took place at 37 °C with continuous shaking at 180 rpm for 16 h, after which glycerol stocks (1 mL) were generated. Expression trials were conducted at temperatures of 15–30 °C for durations of 24–6 h, respectively, with IPTG concentrations varying between 0 and 0.5 mM. A new overnight culture was established by inoculating 1 mL of the glycerol stock into 50 mL of 2×YT media, enhanced with 100 µg·mL^− 1^ ampicillin, and incubating at 37 °C with continuous shaking at 180 rpm for 16 h. This culture was then diluted 1:50 with 2×YT media, enriched with 100 µg·mL^− 1^ ampicillin, and incubated at 37 °C with continuous shaking at 180 rpm until the OD_600_ reached approximately 0.5. Cold shock (4 °C for 20 min) was applied, and overexpression was initiated with 0.5 mM isopropyl-β-D-1-thiogalactopyranoside (IPTG), followed by incubation at 15 °C with continuous shaking at 180 rpm for 24 h. Cells were harvested through centrifugation (5000 × g, 4 °C for 20 min), and the resulting pellet was resuspended in 100 mL of resuspension buffer [10 mM PBS, 0.02% (*w/v*) NaN_3_, pH 7.4] per 1 L of culture. The resuspended pellet was stored at -80 °C overnight. For purification, the cell lysate underwent thawing at 20 °C, followed by sonication for cell lysis. The centrifugation of the cell lysate (18,000 × g, 4 °C for 15 min) aimed to pellet cell debris, isolating the supernatant containing soluble protein.

### Purification of Recombinant *Kp*PrAT

The *Kp*PrAT vector construct incorporates an N-terminal polyhistidine-tag (His-tag), necessitating the use of immobilised metal affinity chromatography (IMAC) for the purification of the recombinant protein. The supernatant underwent sequential passage through 10 mL IMAC Sepharose 6 Fast Flow resin (GE Healthcare, Chicago, Illinois, USA) charged with 0.1 M NiSO_4_ and equilibrated with 10 column volumes of equilibration buffer [10 mM PBS, 0.02% (*w/v*) NaN_3_, 25 mM imidazole, pH 7.2]. This was followed by equilibration buffer, equilibration buffer enhanced with 0.01% (*v/v*) Tween-20, and finally, equilibration buffer. These wash steps were crucial for minimising non-specifically bound proteins to the column. Elution of the recombinant *Kp*PrAT was achieved by passing elution buffer [10 mM PBS, 0.02% (*w/v*) NaN_3_, 500 mM imidazole, pH 7.2]. The purity of the protein was evaluated by employing a glycine SDS-PAGE gel [30]. The spectrophotometric method was employed to measure the protein concentration, utilising a molar extinction coefficient (ɛ) of 92 945 M^− 1^·cm^− 1^. The protein was subsequently dialysed against a sodium phosphate buffer [20 mM Na_2_HPO_4_, pH 7.4], utilising a cellulose membrane dialysis tubing with a molecular weight cut-off of 12 kDa (Sigma-Aldrich, St. Louis, Missouri, USA), for 16 h at 4 °C. The resulting concentration post-dialysis was 24.36 µM.

### Secondary Structure Analysis of Recombinant *Kp*PrAT

Far-UV circular dichroism (CD) spectroscopy analysis was conducted to evaluate the secondary structural composition of *Kp*PrAT. The Jasco J-810 CD spectrophotometer (Jasco, UK) was utilised for this purpose. The protein was subjected to dialysis using sodium phosphate buffer [20 mM Na_2_HPO_4_, pH 7.4] and subsequently stored at 4 °C for 16 h. The native sample contained 2 µM *Kp*PrAT, with or without the presence of 5 mM of the metal salt (MgCl_2_). Spectra were recorded in the wavelength range of 180–260 nm at 20 °C. The mean residue ellipticity [*θ*] was calculated using the formula:

$$\left[\theta \right]=\frac{100\times \theta }{C\times n\times l}$$

in this formula, *θ* represents the ellipticity in millidegree, *C* denotes the concentration of the recombinant protein in mM, the variable *n* is the sum of amino acid residues, and *l* is the pathlength of the cuvette in centimeters. The CONTIN-LL algorithm in Dichroweb was employed to determine the percentage of secondary structural content for *Kp*PrAT in both the absence and presence of the metal salt (MgCl_2_) [31–33].

### Fluorescence Spectroscopy to Assess Substrate Binding Using ANS and mant-ATP

The Jasco FP-6300 fluorometer (Jasco, UK) was used to investigate the binding interactions of 8-Anilino-1-naphthalene (ANS) and nucleotide-binding, 2ʹ/3’-*O*-(*N*-Methylanthraniloyl) adenosine 5ʹ-triphosphate (mant-ATP). Solutions of 1 mM ANS and 1.5 mM mant-ATP were prepared in 20 mM sodium phosphate buffer (pH 7.4). For ANS binding analyses, analyte samples were composed of 2 µM *Kp*PrAT and 0.1 mM ANS. Additionally, the impact of the absence or presence of 0.1 mM ATP and 5 mM MgCl_2_ salt was under observation. Nucleotide-binding studies involved analyte samples containing 2 µM *Kp*PrAT and 10 µM mant-ATP, with the effects of 5 mM MgCl_2_ being monitored. Experimental configurations included a data pitch of 0.5 nm, emission measurement mode, and three accumulations. The excitation wavelength was adjusted to 395 nm or355 nm for ANS and nucleotide-binding, respectively. Spectra were captured within the 400–650 nm range. The experiments were replicated in triplicate at 20 °C.

### Protein Stability Assessment

#### SYPRO Orange Thermal Shift Assay

The thermal stability of *Kp*PrAT was assessed using a CFX96 Touch Real-Time PCR detection system (Bio-Rad, CA, USA). Analyte samples, containing 20 µM *Kp*PrAT and 10 × SYPRO orange fluorescent dye, were prepared in 20 mM sodium phosphate buffer (pH 7.4). The impact of 0.1 mM ATP and 5 mM MgCl_2_ was observed. A reaction volume of 25 µl was loaded onto a CFX96 Touch Real-Time PCR detection system 96-well PCR plate (Bio-Rad, CA, USA). Parameters included a melt curve with a starting temperature range of 10–95 °C and increments of 0.5 °C were observed for 10 s.

#### Thermal Unfolding with Circular Dichroism

The change in the molar ellipticity signal at 222 nm was monitored at increasing temperature. This was performed to assess the impact of temperature, ATP, and MgCl_2_ on the protein’s secondary structural content. The Jasco J-810 spectrophotometer (Jasco, UK), coupled with the Jasco Peltier temperature controller (Jasco, UK), was employed to monitor the protein unfolding process. The protein underwent preparation through dialysis against 20 mM sodium phosphate buffer [20 mM Na_2_HPO_4_, pH 7.4] and was subsequently stored at 4 °C for 16 h. The samples, comprising 2 µM *Kp*PrAT, were prepared with or without 5 mM MgCl_2_ and 0.1 mM ATP. The temperature was incrementally raised from 20 to 70 °C, using a 2 mm quartz cuvette. Computational parameters included a temperature gradient of 2 °C·min^− 1^, data pitch of 0.5 °C, and bandwidth of 2.5 nm.

### Isothermal Titration Calorimetry

The Nano ITC standard volume (TA instruments, USA) was employed to assess the thermodynamic parameters associated with ATP binding to *Kp*PrAT, with or without MgCl_2_. After purification, protein fractions were eluted into 5 mM Ethylenediaminetetraacetic acid (EDTA) at pH 7.2, followed by passage through a PD-10 desalting column packed with Sephadex G-25 resin (Sigma-Aldrich, St. Louis, Missouri, USA) and eluted with sodium phosphate buffer [20 mM Na_2_HPO_4_, pH 7.4]. Subsequently, the protein underwent dialysis against sodium phosphate buffer [20 mM Na_2_HPO_4_, pH 7.4] for 16 h at 4 °C. Both the substrate and protein were prepared with 20 mM sodium phosphate at pH 7.4, followed by degassing. The sample cell was loaded with approximately 20 µM *Kp*PrAT, 1 mM Tris(2-carboxyethyl)phosphine hydrochloride (TCEP), and with or without 5 mM MgCl_2_. The titrated substrate contained 800 µM ATP, 1 mM TCEP, and with or without 5 mM MgCl_2_. Experimental conditions comprised a stirring speed of 250 rpm, a temperature of 293.15 K, 5 µL injections (except for the initial 2 µL injection), 300-second injection intervals, and a total of 20 injections. Following each run, data analysis was conducted using the NanoAnalyze software, corrected utilising the blank (substrate injected into 1 mM TCEP, either with or without 5 mM MgCl_2_), and fitted using the independent model. Consequently, the resulting software analysis provided the Gibbs free energy (*∆G°*), enthalpy (*∆H°*), temperature multiplied by entropy (*T∆S°*), dissociation constant (*K*_d_), and binding stoichiometry (*n*).

### Computational Methods

#### Computer Hardware

Molecular modelling studies were conducted on two high-performance computing units. The Maestro algorithm was utilised on a Windows OS PC with an AMD RYZEN Threadripper 1950X Processor, Asus Rog Strix X399-E Gaming Ryzen AMD, 4 TB internal SSD, 4.0 GHz Precision Boost X399 chipset, 64GB DDR4 RAM, and MSI GeForce RTX 2080 Ti graphics card. The Desmond molecular dynamics (MD) simulation algorithm was employed on an Ubuntu OS PC with an AMD Threadripper 3990X, MSI TRX40 PRO 10G motherboard, GeForce RTX 2070 graphics card, 64GB RAM, 1 TB M.2 SSD, and a 4 TB HDD.

#### Homology Modelling of *Kp*PrAT, Protein and Ligand Preparation

The *Kp*PrAT sequence (UniProtKB accession B5XQE2) was retrieved from UniProt and used in Swiss-Model for homology modelling. The most suitable template (PDB: 6K20) from an *E. coli* homologue, with a sequence identity of over 78%, was employed to construct the *Kp*PrAT homology model. ProCheck [34] was then employed to assess the stereochemical properties of the model. The homology model underwent preparation using the protein preparation wizard module in Maestro v12.2. The pre-processing steps involved assigning bond orders, adding hydrogen atoms, adjusting bond orders for metals and disulfide bonds, and removing water molecules within a 5 Å radius from heterogeny atoms. Optimisation of the hydrogen bonding network was achieved by sampling water molecule orientations using the PROPKA algorithm at pH 7.0. Subsequent refinement involved minimisation using the OPLS_2005 force field, with restraints placed on heavy atoms. The minimisation process concluded when heavy atoms converged using a RMSD cutoff of 0.3 Å. Side chain stereochemistry was checked to ensure minimal perturbations. The finalised, minimised structure was saved as a Maestro (.mae) file for further analysis.

#### Induced Fit Ligand Docking

Induced fit ligand docking, conducted with Schrödinger Maestro v12.2, was used to predict ATP binding to *Kp*PrAT. This method was chosen due to its ability to consider conformational changes in the protein structure induced by ligand binding. An implicit solvent model and the OPLS_2005 force field were applied during the process. The protocol involved ring conformational sampling, with a 2.5 kcal·mol^− 1^ energy barrier and a non-planar conformation penalty on amide bonds. Receptor and ligand scaling was set at 0.5, allowing a maximum of 20 poses per ligand. Residues within a 5.0 Å radius of the docked ligand underwent further refinement using the Prime Refinement algorithm in Maestro v12.2. The Prime energy algorithm was then used to rank the refined protein-ligand complexes. To ensure robust results, a final round of Glide docking and scoring was performed on the receptor structure within 30.0 kcal·mol^− 1^ of the minimum energy structure. In this step, each ligand was re-docked into every refined low-energy receptor structure using default Glide XP settings.

#### Molecular Dynamic Simulations

MD simulations were conducted using the Desmond molecular dynamics simulation engine, integrated into Maestro v12.2. The systems for Apo_*Kp*PrAT and *Kp*PrAT: ATP’s top-scoring poses were saved as .mae files and transferred to a Linux (Ubuntu) desktop server for Desmond MD simulations. Before the simulations, the four systems (Apo_*Kp*PrAT, *Kp*PrAT: Mg^2+^, *Kp*PrAT: ATP, and *Kp*PrAT: Mg^2+^:ATP) were constructed with the Desmond System Builder module. This included solvating with TIP3P-explicit solvent and employing the OPLS_2005 force field. The Apo_*Kp*PrAT or *Kp*PrAT-ligand complex structure was placed within an orthorhombic box, and counter ions were added for system neutralisation. To mimic physiological conditions, 0.15 M NaCl or MgCl_2_ was added. The MD simulation comprised eight stages, with stages 1 to 7 for equilibration and stage 8 for the final 250 ns long-range simulation. Stage 1 determined system parameters, while stage 2 involved a 100 ps simulation using Brownian Dynamics under NVT conditions at 10 K. Stage 3 included a 12 ps simulation under NVT conditions at 10 K. Stages 4, 6, and 7 used short simulation steps under NPT conditions at 10 K, applying restraints on heavy atoms for stages 4 and 6. The final MD stage was conducted at a constant temperature of 300 K.

#### Post Dynamic Analysis

Post-dynamic analyses of MD simulation trajectories were conducted using Schrödinger Maestro v12.2 and Bio3D R-Statistical package for comparative protein structure analysis. Analyses included: (1) Simulation Quality Analysis: The quality of the simulations, which includes parameters such as average energy, pressure, temperature, and volume of each simulated system, was assessed using the Simulation Quality Analysis tool, which is integrated into Maestro v12.2. (2) Structural Analysis: (i) Root-Mean-Square-Deviation (RMSD) of the alpha carbon atoms (C_α_) was computed to evaluate the structural deviation of the protein over the course of the simulation. Additionally, RMSD of the ligand with respect to the receptor was calculated to understand the ligand’s binding stability. (ii) Root-Mean-Square Fluctuations (RMSF) values of residues were analysed to determine their flexibility and motion throughout the simulation, and (iii) protein-ligand Interaction analysis. The following analyses were performed using the Simulation Interaction Diagram algorithm, which is integrated into Maestro v12.2. (v) Radius of Gyration (R_g_), its calculations were carried out to measure the compactness of the protein structure at different time points in the simulation. (vi) Atomic Distance Calculations, which are distances between specific atomic pairs or groups, were computed to investigate molecular interactions. Cα RMSD trajectory clustering in Desmond identified prevalent protein conformations or states during the simulation.

## Results

### Expression and Purification of Recombinant *Kp*PrAT

**Fig. 2:**
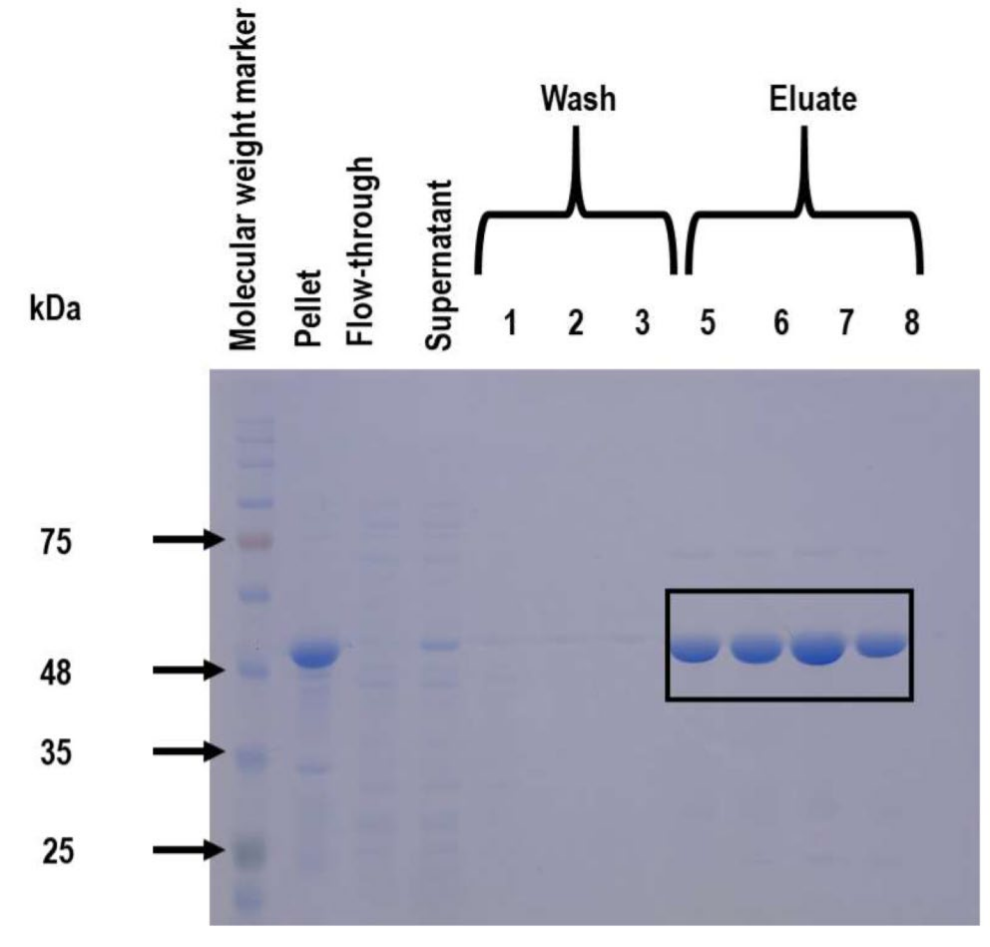
A 12.5% (*w/v*) SDS-PAGE gel for the analysis of expression and purification of *Kp*PrAT. The expression of recombinant *Kp*PrAT was induced with 0.5 mM IPTG (15 °C, 180 rpm, for 24 h). Centrifugation was used to separate the insoluble fraction (pellet) from the soluble fraction (supernatant). The supernatant was then passed through a Ni^2+^-IMAC column, and non-specifically or weakly bound protein (flow-through) was then disadsorbed from the column. The column was then subjected to 3 wash steps to eliminate contaminants. A single-step elution with 500 mM imidazole was used to elute the protein. Following purification, a 12.5% (*w/v*) separating gel was then employed to analyse the quality of expression and purity. A Coomassie blue stain was used to visualise the constituents of the gel. The eluate fractions are boxed and have a predicted molecular weight of ∼ 54 kDa

The pET-11a expression vector (refer to Figure S1) successfully facilitated the expression of recombinant *Kp*PrAT in T7 *E. coli* cells. Expression trials were conducted at 15–30 °C for 24–6 h, respectively, with varying IPTG concentrations at 0 or 0.5 mM. Solubility assessment of the protein was performed using a 12.5% (*w/v*) SDS-PAGE gel (see Figure S1). Optimal conditions for recombinant *Kp*PrAT expression were determined to be 0.5 mM IPTG, 15 °C, 24 h, and 180 rpm. Following expression, Ni^2+^-IMAC was utilised to assess purity. A 12.5% (*w/v*) SDS-PAGE gel (refer to Figure 2) visually depicted the quality of purity. The protein, with a theoretical molecular weight of approximately 54 kDa, eluted just above 48 kDa, highlighted in a black box. Spectrophotometry determined the concentration to be around 0.85 mg·mL^− 1^ (25 mL containing approximately 21.25 mg of pure recombinant *Kp*PrAT from a 1 L culture).

### Secondary Structural Analysis of Recombinant *Kp*PrAT

**Fig. 3:**
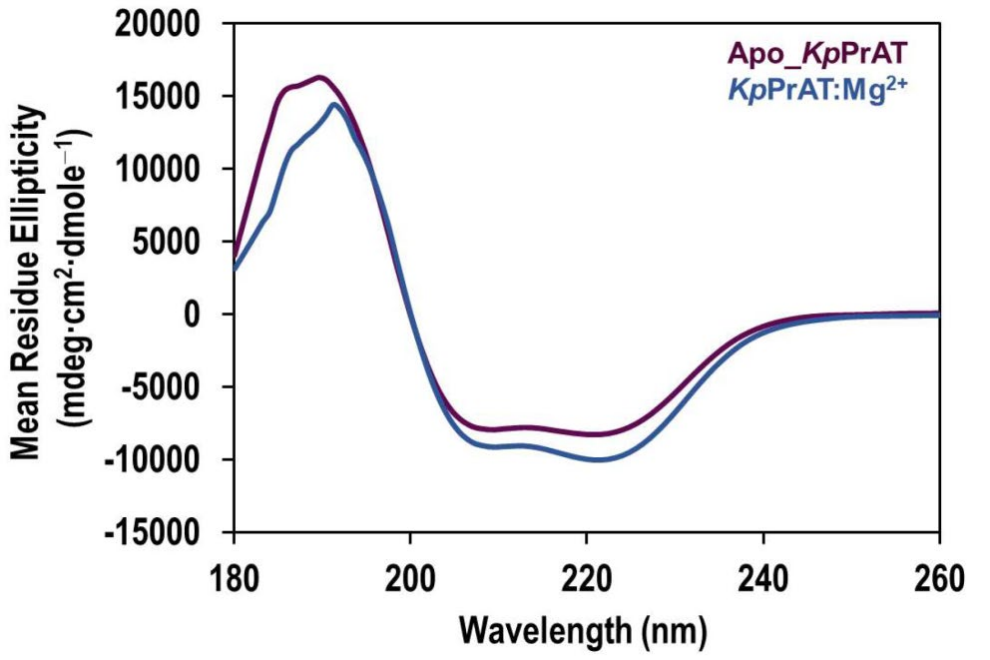
Depicts the Far-UV CD spectra of *Kp*PrAT. Both apoprotein and protein in the presence of Mg ions were prepared in 20 mM sodium phosphate buffer (pH 7.4). The recombinant protein concentration used was 2 µM, with the addition of 5 mM MgCl_2_. The experimental setup included a 0.2 cm path length, 2.5 nm bandwidth, and 0.2 nm data pitch. Each spectrum is an average of 3 replicates, and data smoothing was performed with SigmaPlot v12. Spectra were recorded between 180–260 nm at 20 °C, revealing two troughs at 208 and approximately 220 nm

The secondary structural content of the native state of *Kp*PrAT was assessed with far-UV CD spectroscopy. The resulting spectrum of apoprotein indicates two troughs at 208 and ∼ 220 nm, with a positive peak at ∼ 187 nm (Figure 3). The presence of MgCl_2_ illustrates two troughs at 208 nm and ∼ 220.50 nm. This is indicative of a predominantly α-helical protein both in the absence or presence of MgCl_2_. The fraction of secondary structural content was further assessed with Dichroweb in conjunction with the CONTIN-LL algorithm and data set 4 (Table 1).

**Table 1:** The effect of Mg^2+^ on the secondary structure of *Kp*PrAT

	α-Helix	β-Strand	β-Turn	Unordered	NRMSD^*^
Apo_*Kp*PrAT	0.24	0.14	0.22	0.32	0.06
*Kp*PrAT: Mg^2+^	0.21	0.27	0.19	0.33	0.08

^*^Normalised root mean square deviation (NRMSD) is a statistical value indicative of the fit of the experimental data relative to the predicted data. Values less than 0.1 represent a better fit

### Fluorescence Spectroscopy for ANS Binding and mant-ATP Binding to Recombinant *Kp*PrAT

**Fig. 4:**
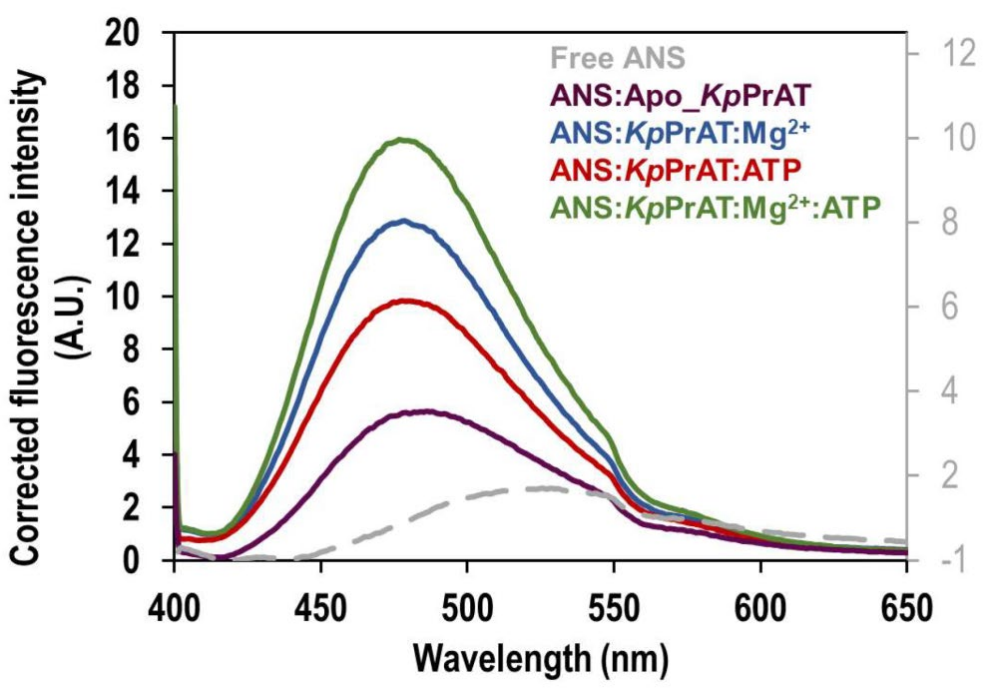
Displays the ANS binding spectra to *Kp*PrAT for assessing substrate binding. The influence of 0.1 mM ATP and 5 mM MgCl_2_ on ANS fluorescence emission was examined. The analytes, in conjunction with 2 µM *Kp*PrAT, were prepared in 20 mM sodium phosphate buffer (pH 7.4). Excitation of the fluorescent probe occurred at 395 nm, and emission was observed within the 400–650 nm range

Extrinsic fluorescence spectroscopy was employed to assess the protein’s tertiary structure. Hydrophobic fluorescent probes, ANS and mant-ATP, were used to characterise nucleotide binding to recombinant *Kp*PrAT. Monitoring changes in the maximum emission wavelength and fluorescence intensity, the assessment revealed ANS binding to a hydrophobic site by detecting a blue shift in the maximum emission wavelength and an increase in fluorescence intensity. Figure 4 illustrates the impact of MgCl_2_ on ANS fluorescence emission. Table 2 details the shift type and difference in maximum emission wavelength of analytes relative to free ANS. ANS-bound protein exhibited increased fluorescence intensity and a blue shift compared to free ANS, indicating the presence of ANS binding pockets on *Kp*PrAT. Similar observations of a blue shift and increased fluorescence intensity were noted in the presence of ATP, with and without Mg^2+^. However, a higher fluorescence emission of ANS was observed in the presence of ATP with Mg^2+^ compared to ATP without Mg^2+^. The response of ANS is influenced by the binding site’s polarity and hydrophobicity [35]. The association of ATP and Mg^2+^ enhances hydrophobicity in the binding pocket through coordinated interaction, potentially releasing water from the hydrophobic pocket. This heightened hydrophobicity leads to an increased quantum yield, serving as an index of polarity. These findings suggest a conformational change upon ATP and Mg^2+^ binding to *Kp*PrAT. Figure S3 and S4 presents the spectra of the replicates of ANS and nucleotide emission, respectively.

**Table 2:** Extrinsic ANS fluorescence maximum emission intensity, maximum emission wavelength (nm), shifts in the maximum emission wavelength, and the direction and type of shift

	Maximum fluorescence intensity	λ_max_ (nm)	Δ in λ_max (Bound−Free ANS)_	Shift type
Free ANS	1.20 ± 0.05	525.00		
ANS: Apo_*Kp*PrAT	5.25 ± 0.86	486.00	36.00	Blue
ANS:*Kp*PrAT: ATP	9.44 ± 3.61	477.50	47.50	Blue
ANS:*Kp*PrAT: Mg^2+^	12.48 ± 1.78	478.50	46.50	Blue
ANS:*Kp*PrAT: Mg^2+^:ATP	15.56 ± 2.96	476.50	48.50	Blue

**Fig. 5:**
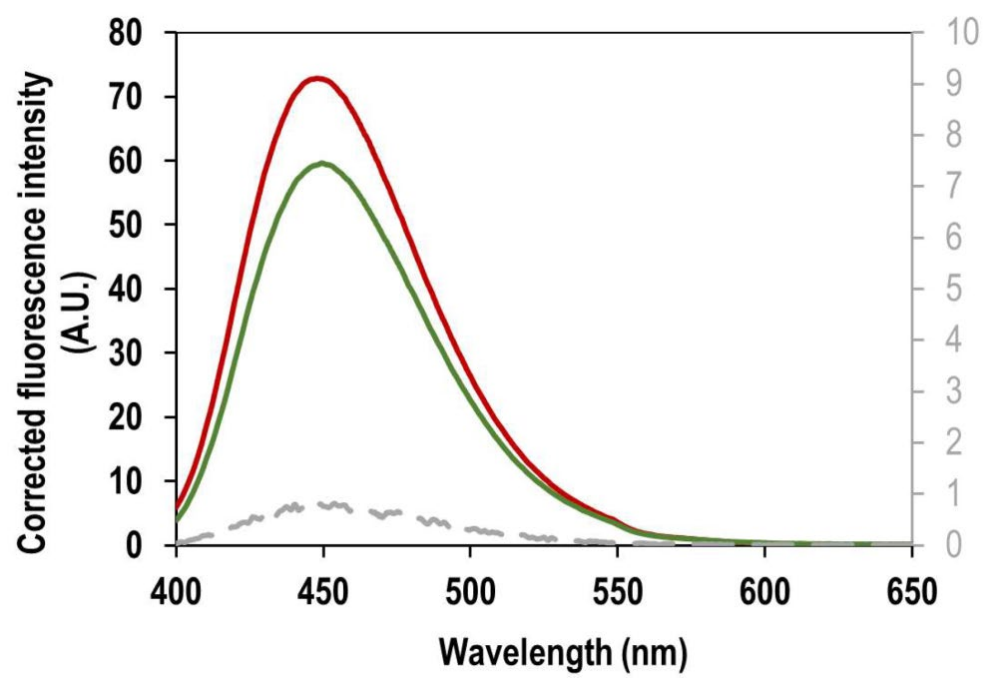
Spectra of mant-ATP binding to *Kp*PrAT. The effects of 10 µM mant-ATP, and 5 mM MgCl_2_ on binding to 2 µM *Kp*PrAT were monitored. The analytes were prepared in 20 mM sodium phosphate buffer (pH 7.4). Excitation of the fluorescent nucleotide occurred at 355 nm, and emission was observed within the 400–650 nm range. The grey spectrum is free mant-ATP, while the red and green spectra represent the protein bound to ATP without and with Mg^2+^, respectively

Mant-ATP, fluorescent nucleotide, selectively bind to the ATP-binding site of ATP-binding proteins. Successful binding is confirmed by a decrease in the maximum emission wavelength (blue shift) and an increase in fluorescence intensity. Figure 5 depicts fluorescence spectra of mant-ATP bound to *Kp*PrAT, with or without Mg^2+^. Table 3 details changes in maximum emission wavelength, fluorescence intensity, and shift characteristics. An increase in fluorescence intensity and blue shift occurs when *Kp*PrAT binds to mant-ATP, irrespective of Mg^2+^ presence, indicating its role as an ATP-binding protein.

**Table 3:** Mant-ATP binding to *Kp*PrAT, either with or without of Mg^2+^

	Maximum fluorescence intensity	λ_max_ (nm)	Δ in λ_max (Free−Bound mant−ATP)_	Shift type
Free mant-ATP	0.82 ± 8.67	453.50		
Mant-ATP:*Kp*PrAT	72.90 ± 8.93	448.00	5.50	Blue
Mant-ATP:*Kp*PrAT: Mg^2+^	59.70 ± 1.35	449.50	4.00	Blue

### Thermal Stability Studies of Recombinant *Kp*PrAT

#### SYPRO Orange Thermal Shift Assay

**Fig. 6:**
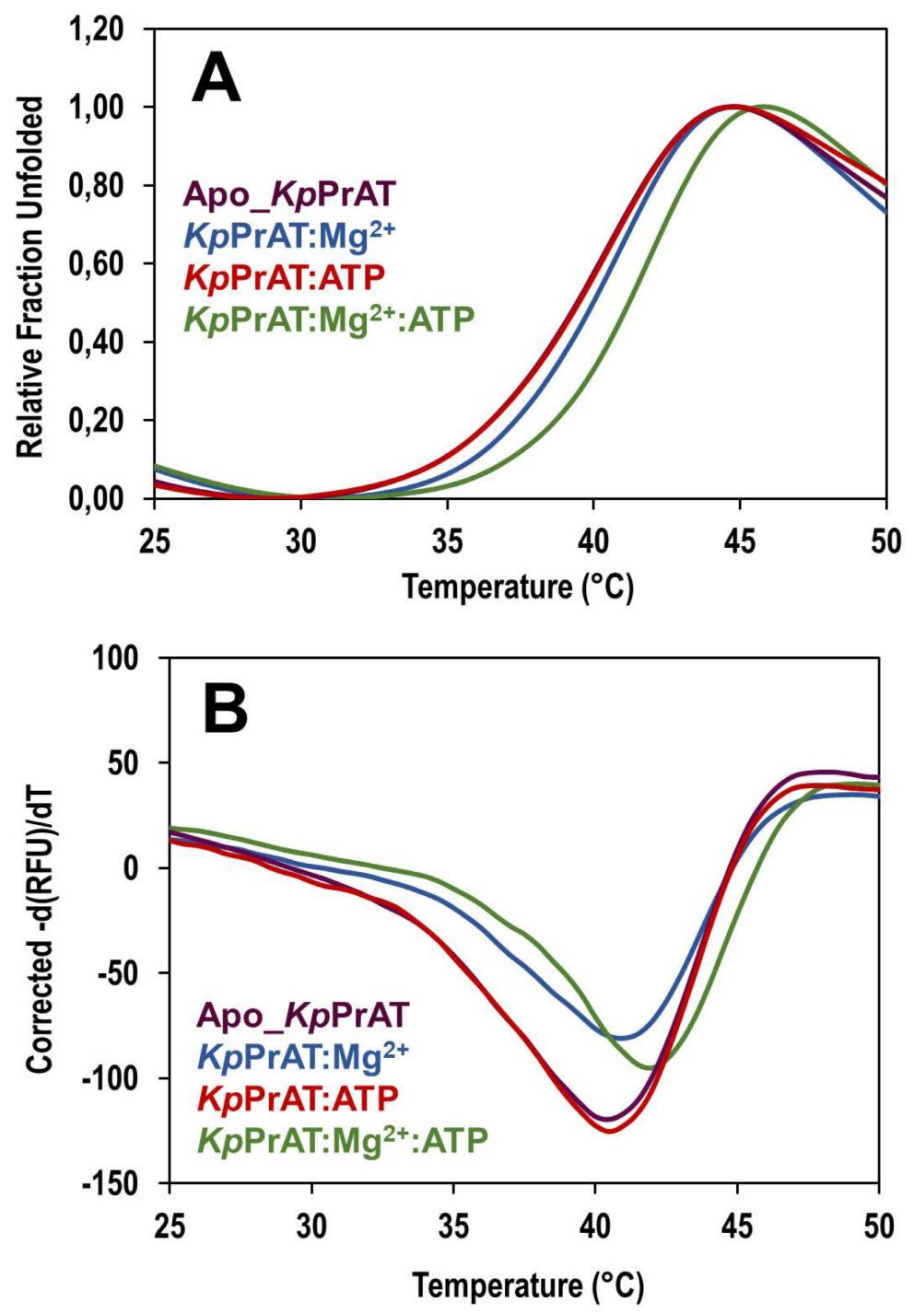
Melt curves of the unfolding of *Kp*PrAT. The effects of 0.1 mM ATP and 5 mM MgCl_2_ on binding to 20 µM *Kp*PrAT were monitored. The analytes, including 10 × SYPRO orange, were prepared in 20 mM sodium phosphate buffer (pH 7.4). (**A**) Fluorescence emission spectra in fraction unfolded of the dye, measured against a temperature range of 25–50 °C. (**B**) The melting curves of the protein were obtained under conditions with or without ATP or Mg^2+^

The thermal unfolding of *Kp*PrAT was monitored to assess its thermal stability. The melting temperature (*T*_m_) was derived from the protein’s melting curve under conditions with or without ATP or Mg ion (Figure 6). A 0.5 °C increase in *T*_m_ is noted when the protein is bound to Mg^2+^ compared to the apoprotein. Additionally, the *T*_m_ reaches its maximum in the presence of both ATP and Mg^2+^, surpassing values for the apoprotein or protein bound to ATP alone (Table 4). These findings suggest an improved thermal stability of the protein when bound to both ATP and Mg^2+^ compared to the apoprotein. Figure S5A and S5B presents the melting curves of the replicates of SYPRO orange thermal unfolding.

**Table 4:** The influence of ATP and Mg^2+^ on the thermal unfolding of *Kp*PrAT was investigated. The protein’s thermal stability was assessed by determining the melting temperature (*T*_m_) in °C

	Melting temperature (*T*_m_ in °C)	
	Absence of Mg^2+^	Presence of Mg^2+^
Apo_*Kp*PrAT	40.50 ± 0.00	41 ± 0.29
*Kp*PrAT: ATP	40.50 ± 0.00	42 ± 0.00

#### Thermal Unfolding with Circular Dichroism

**Fig. 7:**
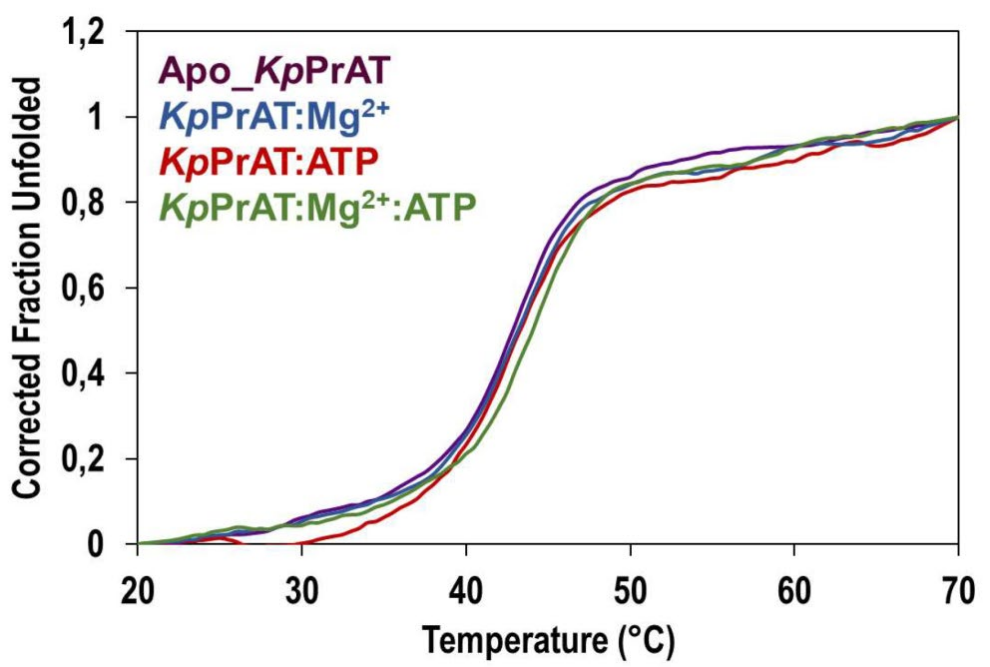
An illustration of the thermal unfolding curves of *Kp*PrAT using circular dichroism. The samples were prepared in a solution of 20 mM sodium phosphate buffer [20 mM Na_2_HPO_4_, pH 7.4]. The mean residue ellipticity was monitored at 222 nm for a concentration of 2 µM *Kp*PrAT, and the effects of 5 mM MgCl_2_ and 0.1 mM ATP were observed. The temperature ranged from 20 to 70 °C was analysed and raw data were subjected to fitting using the smoothers 2D tool within SigmaPlot v12

Thermal stability was assessed using far-UV CD spectroscopy at 222 nm. The melting curves obtained by plotting the mean residue ellipticity at 222 nm (mdeg·cm^2^·dmole^− 1^) against temperature (°C) provided the *T*_m_, serving as an indicator of the protein’s thermal stability (Figure 7 and S5C). A decrease of 0.33 °C in the *T*_m_ is observed when ATP binds to the protein alone compared to the apo protein. Conversely, the presence of the divalent metal ion alone results in a 0.17 °C increase in the *T*_m_. The most significant increase in the *T*_m_ (Table 5) occurs when both ATP and Mg^2+^ are bound to the protein. These findings align with those of the SYPRO orange thermal shift assay, indicating that the complex of ATP, Mg^2+^, and *Kp*PrAT improves the protein’s thermal stability.

**Table 5:** The effects of ATP and Mg^2+^ on the thermal stability of *Kp*PrAT was investigated. The thermal stability of the protein is quantified by its melting temperature (*T*_m_), measured in °C

	Melting temperature (*T*_m_ in °C)	
	Absence of Mg^2+^	Presence of Mg^2+^
Apo_*Kp*PrAT	42.83 ± 0.29	43 ± 0.50
*Kp*PrAT: ATP	42.50 ± 0.87	44 ± 0.50

### Thermodynamics of ATP Association with *Kp*PrAT

**Fig. 8:**
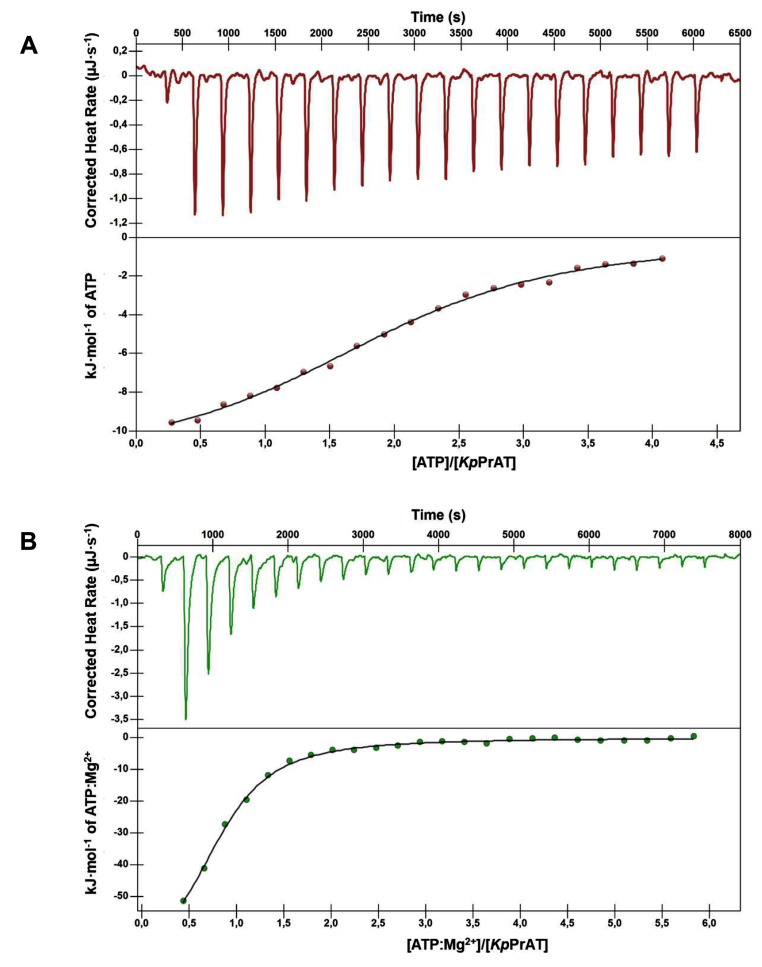
Thermograms depicting the binding of ATP to *Kp*PrAT under two conditions: (**A**) in the absence of Mg^2+^, and (**B**) in the presence of Mg^2+^, were generated. Isothermal titration calorimetry (ITC) was employed to investigate the interaction between 800 µM ATP and approximately 20 µM *Kp*PrAT, both prepared in sodium phosphate buffer [20 mM NaH_2_PO_4_, pH 7.4]. Samples were supplemented with 1 mM TCEP and either with or without 5 mM MgCl_2_. Data analysis was conducted using NanoAnalyze software, using the independent model to fit the data. Each figure presents an overlay of the raw titration data in the upper panel, and the corresponding fitted data in the lower panel. In the absence of Mg^2+^, the binding stoichiometry (*n*) was approximately 2, whereas in the presence of the divalent metal ion, it was approximately 1

Isothermal titration calorimetry (ITC) was employed to ascertain the thermodynamic parameters of ATP binding to *Kp*PrAT, with the influence of MgCl_2_ being monitored. The interaction between ATP and *Kp*PrAT resulted in an exothermic reaction, evidenced by the negative *∆H°* (Figure 8A and B). Furthermore, the reaction displayed spontaneity, as indicated by the negative *∆G°*, and exhibited a stoichiometry of 1 ATP molecule per mole of *Kp*PrAT, in the presence of Mg^2+^, while in the absence of the divalent metal ion, the stoichiometry shifted to 2 ATP molecules per mole of *Kp*PrAT. The thermodynamic parameters are summarised in Table 6.

**Table 6:** Thermodynamic parameters of the interaction of ATP and *Kp*PrAT, either with or without Mg^2+^

	*K*_d_ (µM)	∆*H*° (kJ·mol^− 1^)	T∆*S*° (kJ·mol^− 1^)	∆*G*° (kJ·mol^− 1^)
*Kp*PrAT: ATP	4.02	-11.85	18.44	-30.28
*Kp*PrAT: Mg^2+^:ATP	2.93	-76.92	45.87	-31.05

### Molecular Modelling Studies

The Swiss modelling tool was used to build a homology model of *Kp*PrAT, using the *E. coli* protein adenylyltransferase homologue (PDB:6K20) as a template. Of the 468 amino acid residues on the template, only 465 amino acid residues of the homology model aligned to the template, which resulted in a root-mean-squared deviation (RMSD) of 0.062 Å, with PyMol. Furthermore, the model had a Global Model Quality Estimate (GMQE) and Qualitative Model Energy Analysis Distance Constraints (QMEANDisCo) of 0.94 and 0.90, respectively (Figure S6). These parameters are an indicator of the quality of the model and are represented as values between 0 and 1. The higher the value, the higher the quality. This suggests that the quality of the homology model is high. Furthermore, the homology model was compared to an AlphaFold model, and the alignment conducted in PyMol revealed an RMSD of 0.33. Subsequently, both models were validated using PDBsum. The resulting Ramachandran plots indicated that at least 90% of residues in both models fell within the allowed region (Figure S6). The sequence alignment of *Kp*PrAT and *E. coli* adenylyltransferase show a sequence identity of 78.45%, thus the template is suitable for building the homology model of *Kp*PrAT (Figure S2 and S6). The Ramachandran analysis show that there were 95.92% of residues in the favoured region, and 0% of residues in the disallowed regions (Figure S6).

The highest-scoring poses and apo-structure were subjected to additional analysis through the Desmond molecular dynamics simulation, utilising Maestro v12. The average, standard deviation and slopes of the total and potential energies, temperature, pressure, and volume were recorded for all four systems (Table 7). The slopes of each system were ∼ 0 ps^− 1^, thus suggesting that there was complete equilibration before the commencement of the 250 ns simulations.

**Table 7:** The analysis of simulation quality was performed for the four simulated systems: Apo_*Kp*PrAT, *Kp*PrAT: Mg^2+^, *Kp*PrAT: ATP, and *Kp*PrAT: Mg^2+^:ATP. The table presents the average, standard deviation (SD), and slopes of the total energy (*E*_t_), potential energy (*E*_p_), temperature (*K*), pressure (*P*), and volume (*V*) of the systems throughout a 250 ns simulation period

Simulated system	Apo_*Kp*PrAT		*Kp*PrAT: Mg^2+^		*Kp*PrAT: ATP		*Kp*PrAT: Mg^2+^:ATP	
Parameter	Average (± SD)	Slope (ps^− 1^)	Average (± SD)	Slope (ps^− 1^)	Average (± SD)	Slope (ps^− 1^)	Average (± SD)	Slope (ps^− 1^)
*E*_t_ (kcal·mol^− 1^)	-139889.55 (107.74)	-0.00	-158913.31 (103.28)	-0.00	-140185.60 (103.04)	-0.00	-159396.97 (108.43)	-0.00
*E*_p_ (kcal·mol^− 1^)	-171657.54 (95.23)	-0.00	-190654.41 (90.57)	-0.00	-171878.12 (90.14)	-0.00	-191096.02 (95.92)	-0.00
*K*	298.74 (0.46)	0.00	298.73 (0.46)	-0.00	298.74 (0.46)	0.00	298.73 (0.46)	-0.00
*P* (bar)	1.05 (46.64)	-0.00	0.98 (47.34)	-0.00	0.83 (46.80)	-0.00	0.75 (47.51)	-0.00
*V* (Å^3^)	513900.29 (451.36)	-0.00	512127.58 (442.15)	0.00	512228.68 (450.55)	0.00	510944.44 (443.08)	-0.00

**Fig. 9:**
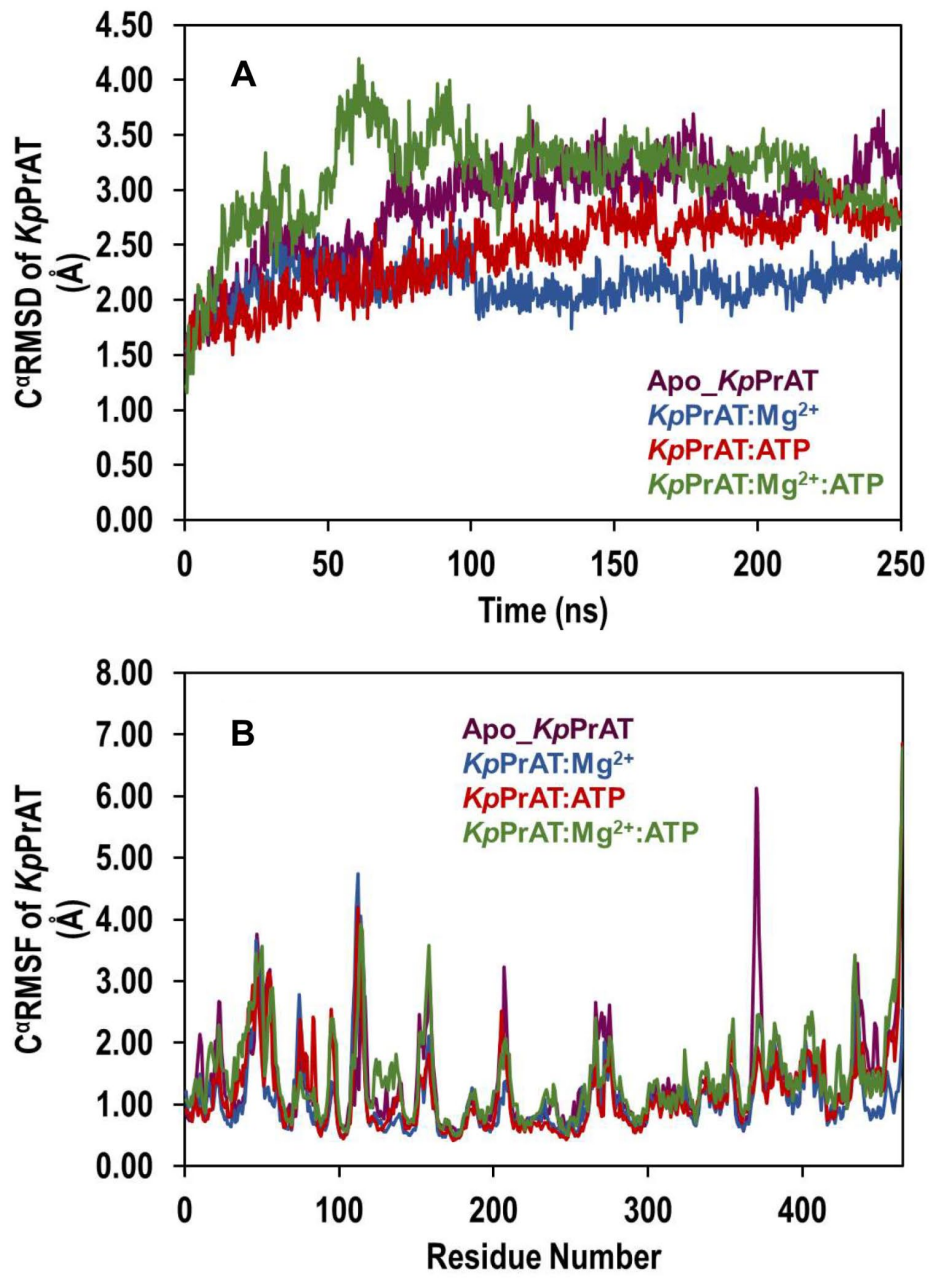
Trajectory analysis was conducted to assess the (**A**) root-mean-square deviation (RMSD) and (**B**) root-mean-square fluctuation (RMSF) of *Kp*PrAT’s Cα atoms during a 250 ns simulation. The systems included Apo_*Kp*PrAT, *Kp*PrAT in complex with MgCl_2_, ATP, or both substrates

To assess the dynamics of *Kp*PrAT as a result from ATP or MgCl_2_ binding, the Cα RMSD, Cα RMSF and radius of gyration were monitored. A variation in the Cα RMSD was recorded for all the systems over the 250 ns simulation. There was a 0.9 and 1.1 Å for *Kp*PrAT: ATP and Apo_*Kp*PrAT Cα RMSD deviation, respectively. While the lowest (0.8 Å) and highest (1.2 Å) deviations were observed for *Kp*PrAT: Mg^2+^ and *Kp*PrAT: Mg^2+^:ATP, respectively (Figure 9A and Figure S7). The presence of ATP and MgCl_2_ results in a system which is dissimilar in conformation compared to the presence of only MgCl_2_. The Cα RMSF provides insights into the flexibility of Cα atoms within the protein, as depicted in Figure 9B. Notably, regions analysed (residue numbers 120–159 and 221–292, as shown in Figure S8) exhibit distinct variations in Cα RMSF, with the region spanning 221–292 showing the most significant fluctuations. The systems involving ATP only and Mg^2+^ only display comparable Cα RMSF throughout the 250 ns simulation, indicating an influence of ATP and the divalent metal ion on protein dynamics. The RMSD of the ligand relative to *Kp*PrAT was assessed over the 250 ns simulation (Figure S9), revealing a substantial change in RMSD in the presence of ATP only compared to Cα RMSD in the same condition. The presence of Mg^2+^ in the complex stabilised ATP throughout the simulation.

**Fig. 10:**
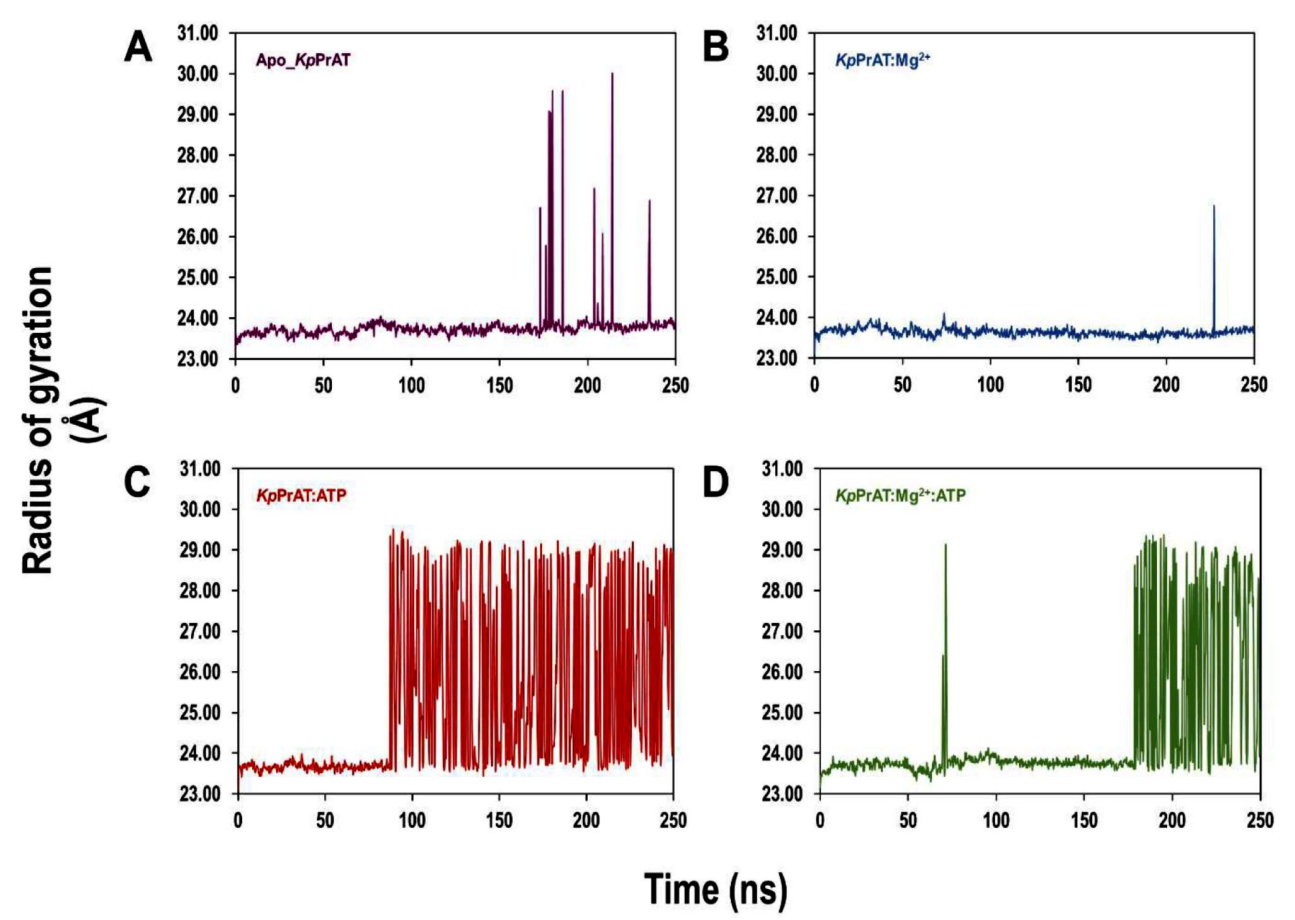
The radius of gyration (R_g_) of the Cα atoms of (A) Apo_*Kp*PrAT, *Kp*PrAT complexed with (B) Mg^2+^, (C) ATP, and (D) ATP and Mg^2+^ over a 250 ns simulation time

The radius of gyration (R_g_) is a measure of compactness of the protein (Figure 10). There is an observed increase in the R_g_ at approximately 90 ns, which is sustained throughout the simulation time, in the presence of ATP only. Nevertheless, an initial rise in the radius of gyration (R_g_) at around 70 ns, succeeded by a decline until 180 ns, is observed when the protein forms a complex with both ATP and Mg^2+^. These findings suggest a decrease in compactness or an increase in the extended structure of the protein, particularly towards the end of the simulation period, upon ATP binding.

**Fig. 11:**
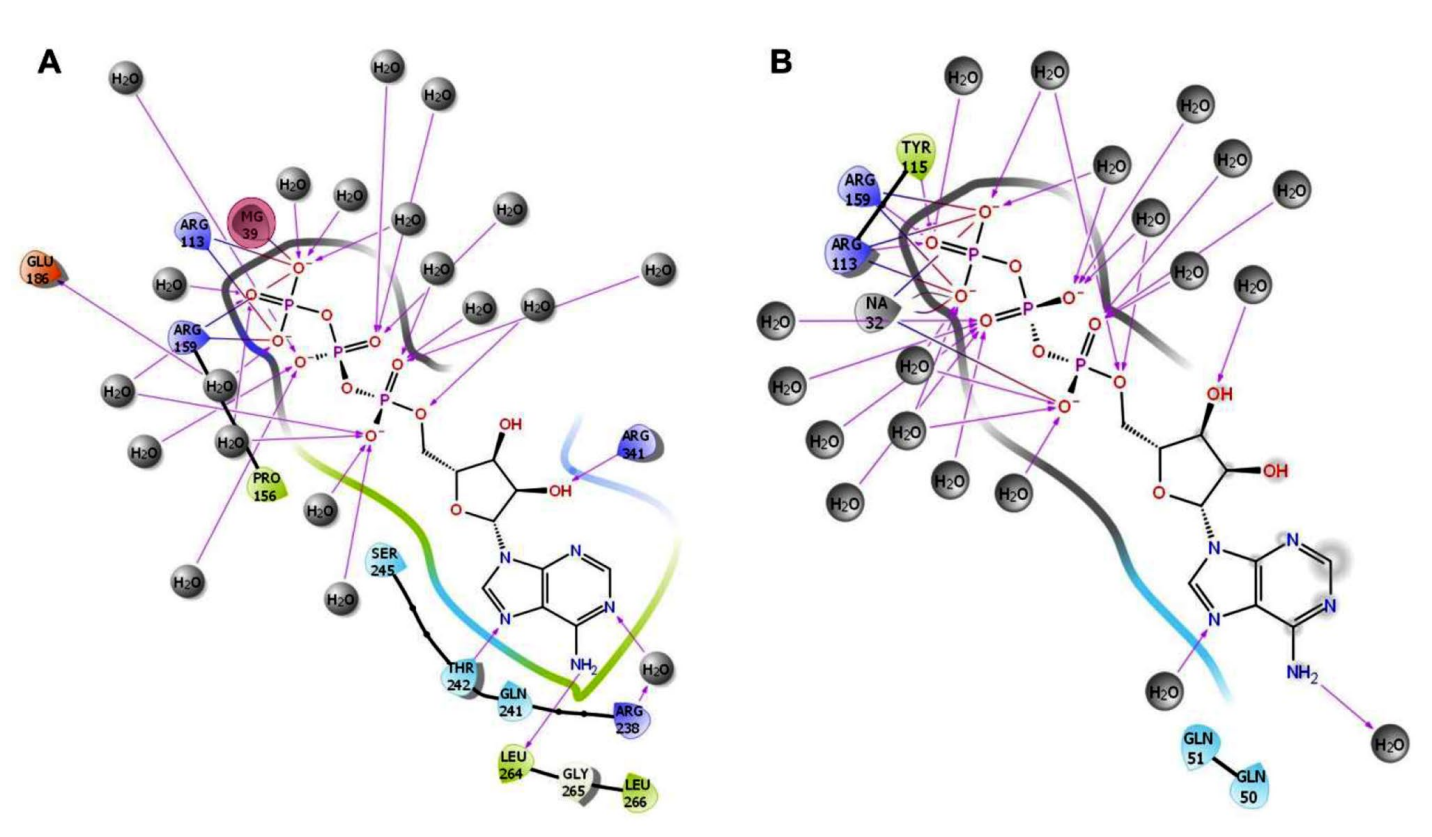
Illustrates a 2D interaction plot displaying clusters of trajectory frames based on the RMSD of the most dominant snapshots of *Kp*PrAT: ATP, either in the (**A**) presence of Mg^2+^ or (**B**) absence of the divalent cation. Amino acid residues within 4 Å of the ligand are represented, with green indicating non-polar amino acids and the hydrophobic region, while polar regions and amino acids are depicted in blue. Grey circles represent either NaCl or water molecules, and grey lines indicate metal coordination. H-bonds are displayed as purple lines. Magnesium 39 is enclosed in a pink circle and forms a salt bridge (blue-red line) with the γ-phosphate of ATP. Positively charged and negatively charged amino acids are represented in violet and orange, respectively. The illustration was created with the Maestro 2D interaction diagram feature incorporated in Schrödinger Maestro v12.2

**Fig. 12:**
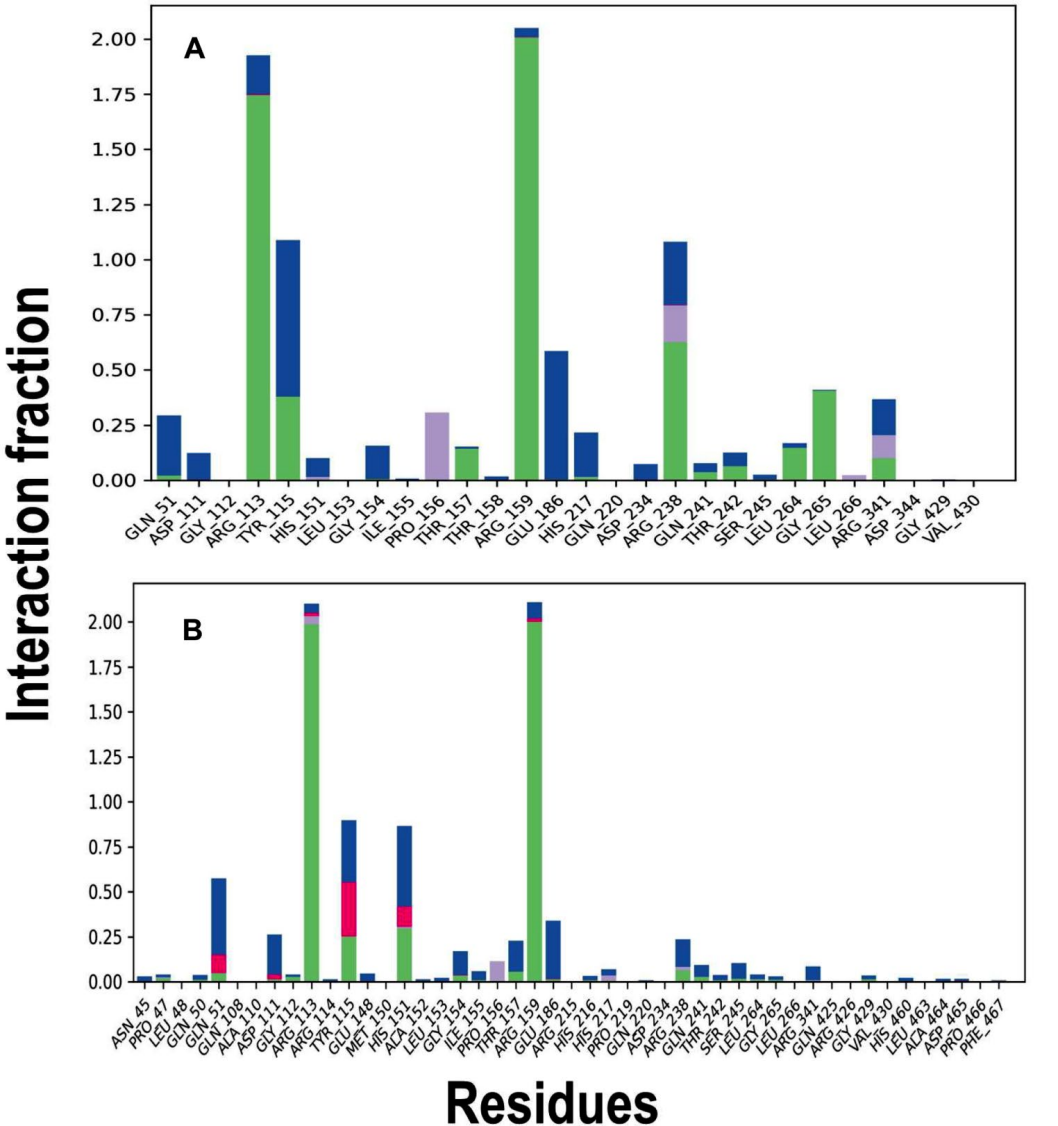
Bar charts displaying stacked representations of side chain interactions and the respective interaction types between *Kp*PrAT: ATP, either in the (**A**) presence of Mg^2+^ or (**B**) absence of Mg^2+^, throughout the 250 ns simulation period. H-bonds are displayed as green, while hydrophobic, ionic interactions and water bridges are represented as grey, pink and blue, respectively. The illustration was created with the Ligand Interaction algorithm feature incorporated in Maestro v12.2

The interaction of ATP with *Kp*PrAT, with or without the divalent metal ion, was examined through 2D interaction plots (Figure 11) and stacked bar charts illustrating side chain interactions (Figure 12). Arg 113 and 159 consistently formed salt bridge interactions with the γ-phosphate, regardless of the presence of the divalent ion. Various interactions, including water bridges, H-bonds, and ionic interactions, were observed, with water bridges being the primary type. Magnesium 39 participated in a salt bridge with the γ-phosphate, indicating metal coordination in this region. In the presence of the divalent ion, an increase in H-bonds occurred, involving the hydroxyl group of the ribose moiety with Arg 341 and the amino group in the adenosine with Leu 264. This interaction resulted in the creation of a hydrophobic region. Additionally, Mg^2+^ introduced hydrophobic clefts, contributing to the emergence of a polar region involving Ser 245, Thr 242, and Gln 241. Conversely, without the divalent ion, Gln 50 and 51 are responsible for creating this polar region. Water molecules form hydrogen bonds with phosphates in both conditions. Notably, without Mg^2+^, there is an increase in ionic interactions, particularly in residues Gln 51, Tyr 115, and Met 150. In the presence of Mg^2+^, water bridges and hydrogen bonds dominate. Hydrophobic interactions involving Pro 156 are observed. Arg 113 and 159 exhibits contact with the γ-phosphate of ATP, constituting 42% and 83%, respectively. In the absence of Mg^2+^, the contact duration increases to 98% and 100%, respectively. Additionally, Arg 238 establishes 61% contact with the amine group of the nitrogenous base in ATP (Figure S10).

Maestro’s SiteMap algorithm facilitated the evaluation of potential binding sites on the protein, assessing druggability and hydrophobicity in the presence of substrate compared to its apo form. The primary parameters that were extracted were the SiteScore, D-Score and the volume of the binding sites (in Å^3^). The SiteScore is a numerical value that assesses a protein site’s likelihood to be a ligand-binding pocket, while the D-Score is a measure of druggability and hydrophobicity. A D-Score greater than 0.83 is classified as druggable, and a D-Score less than 0.83 is classified as undruggable [36]. Additionally, a SiteScore cutoff of 0.80 is employed to identify potential ligand-binding sites [37]. Site 1 exhibited the highest SiteScore and D-Score among all the systems (Table 8). The presence of Mg^2+^ increased the number of potential ligand binding sites on the protein, with all SiteScores surpassing the 0.80 threshold. Omitting the SiteScores did not alter the total volume of the protein complexed with Mg^2+^. Moreover, these volumes exceeded those observed when the protein was not bound to the divalent metal ion.

**Table 8:** SiteMap analysis of the 4 simulated systems. The output parameters extracted are the SiteScore, D-Score and Volume (in Å^3^)

Site	Apo_*Kp*PrAT			*Kp*PrAT: Mg^2+^			*Kp*PrAT: ATP			*Kp*PrAT: Mg^2+^:ATP			
	SiteScore	D-Score	Volume (Å^3^)	SiteScore	D-Score	Volume (Å^3^)	SiteScore	D-Score	Volume (Å^3^)	SiteScore	D-Score	Volume (Å^3^)	
1	0.99	0.95	467.51	1.01	0.97	627.69	1.04	0.91	405.43	1.00	1.00	512.44	
2	0.91	0.60	81.98	0.99	1.03	322.08	0.95	0.86	239.07	0.97	0.99	513.13	
3	0.81	0.79	246.96	0.96	0.98	308.70	0.93	0.58	89.87	0.88	0.71	113.19	
4	0.73	0.66	93.98	0.95	0.99	311.10	0.84	0.85	209.23	0.84	0.70	173.22	
5	0.69	0.57	104.61	0.91	0.81	109.42	0.73	0.64	118.33	0.81	0.80	197.57	
**Total**			**995.04**			**1678.99**			**1061.93**			**1509.54**	
**Revised Total**			796.45*			**1678.99**			853.73*			**1509.54**	

^*^The SiteScore cutoff is 0.80. Volumes which had a SiteScore less than the cutoff were omitted from the summation of the revised total volume

## Discussion

Globally, antimicrobial-resistant, *K. pneumoniae* infections have been a critical clinical challenge due to the lack of availability of efficacious antimicrobials [38]. This has called for more attention to exploring new approaches to combating the issue of AMR. *E. coli* adenylyltransferase has recently been demonstrated to function as a pseudokinase, and its connection to oxidative stress has been established through its interaction with glutaredoxin (Grx). The proposed mechanism of the enzyme’s reaction is to transfer an AMP group from ATP to Grx, subsequently producing an AMPylated Grx and an inorganic pyrophosphate [28]. Grx is a small redox enzyme involved in the glutathione-dependent formation of deoxyribonucleotides, a process catalysed by ribonucleotide reductase [39]. Fundamentally, Grx protects cells against oxidative stress, as it reduces mixed disulfides with glutathione [40]. Inducing oxidative stress in cells by targeting pseudokinase would prove to be a promising approach to tackling the problem of AMR in *K*. *pneumoniae*.

The optimised expression conditions (15 °C, 24 h, 0.5 mM IPTG) following expression trials coupled with a 12.5% (*w/v*) SDS-PAGE gel (Figure 2) showed that the pET-11a expression system was effective in expressing the recombinant *Kp*PrAT protein into a soluble fraction. Furthermore, this electrophoretogram was able to show the success of employing Ni^2+^-IMAC for the single-step purification of the protein. The protein eluted slightly above the ∼ 48 kDa standard. It has a predicted theoretical molecular weight of ∼ 54 kDa. The theoretical molecular weight was predicted using the ProtParam ExPasy tool [41]. The fusion tag on the protein was a hexahistidine tag, which was essential in aiding the purification of recombinant *Kp*PrAT. The transitional metal ion (Ni^2+^) is immobilised in the agarose resin. The passing of tagged protein through the column allows for the coordinate bonds between the Ni^2+^ and histidine fusion tag. The imidazole ring in the histidine amino acid acts as an electron donor thus promoting coordinate bond formation with the transitional metal ion [42]. Furthermore, the single-step purification yielded ∼ 1.54 mg of purified protein per gram of wet *E. coli* cells. These expression studies were able to show that recombinant *Kp*PrAT can be overexpressed and purified to homogeneity, which can be used for structural and functional studies.

The overexpression and subsequent purification of recombinant *Kp*PrAT yielded high quantities of pure protein for structural characterisation. The *E. coli* protein adenylyltransferase is a pseudokinase, sharing a structural fold with protein kinases. However, it lacks the glutamates present in the Y/HRD or DFG motifs, which are essential for catalysing kinase activity [28]. The sequence alignment showed a ∼ 78% sequence similarity between *E*. *coli* protein adenylyltransferase (PDB:6K20) and *K. pneumoniae* protein adenylyltransferase, using the Clustal Omega tool (Figure S2). The protein has a C-lobe and N-lobe, characteristic of a protein kinase. The lobes form a deep cleft in the protein referred to as the active site. The N-lobe is comprised of a β-sheet, with 5 β-strands, and the C-lobe is predominantly α-helical. The Far-UV CD and Dichroweb results show a decrease in the mean residue ellipticity of α-helical content when the divalent cation is present. However, the far-UV CD spectra of protein in the absence or presence of Mg^2+^ demonstrate that the protein has two negative troughs at 208 and 220 nm, representative of a primarily α-helical protein (Figure 2). Additionally, the Dichroweb results show that the protein has a combination of all secondary structural content (Table 1). These results are expected as the conformation of the protein contains a combination of all the secondary structural content, which is characteristic of most bacterial SelO proteins [28, 43]. Furthermore, the NRMSD value for protein both in the absence or presence of MgCl_2_ is less than 0.1, indicating that the fit of the experimental and predicted data is a good fit. Hence, solidifying the accuracy of the Dichroweb results. There is a 0.13 increase in the β-strand fraction when Mg^2+^ is introduced to the protein. Structurally, β-strands are an extension of 4 to 10 amino acids with repetitive hydrophobic and hydrophilic residues [44, 45]. Consequently, they promote H-bonding with each other, to form β-sheets, or amino acid side chains that foster these non-covalent interactions [46]. The β-strands are fundamental in substrate binding, and protein catalysis and mark the active conformation of the protein [26].

Extrinsic fluorescence spectroscopy for tertiary structural analysis provides insights into the binding site environment of recombinant *Kp*PrAT. A hydrophobic fluorescent probe, ANS, and fluorescent nucleotide, mant-ATP were both used to assess the effects of substrate binding to protein. The blue shift and increase in the emission wavelength maxima of ANS with ATP and Mg^2+^ (Table 2) present ANS binding. A comparison of the binding pocket environment of *Kp*PrAT, either in the presence of ATP or Mg^2+^, suggest that transitional metal ion increases hydrophobicity at the site. Apart from neutralising the negative charge of ATP, the metal ion establishes coordinate bonds with the aspartate in the DFG motif and the asparagine following the Y/HRD motif [47]. The pronounced decrease in the emission wavelength maximum, coupled with the increased fluorescence intensity of ANS when both ligands are present signifies the decrease in the polarity of the binding pocket. The blue shift and increased fluorescent intensity of mant-ATP, when ATP and Mg^2+^ are present (Table 3), speaks to the ability of the *Kp*PrAT to bind to ATP. These studies effectively emphasised the conformational change in *Kp*PrAT induced with ATP and Mg^2+^. They suggest the presence of a hydrophobic binding pocket and potentially an ATP and Mg^2+^ binding site.

The SYPRO orange thermal shift assay was employed to examine the impact of ATP and Mg^2+^ on the thermal stability of *Kp*PrAT through thermal unfolding. The ATP did not exert any influence on the stability of *Kp*PrAT (Table 4). However, the presence of Mg^2+^ and both ligands showed a 0.5 °C and 1.5 °C increase in thermal stability compared to apoprotein. This trend was observed in protein kinase A [48]. Furthermore, thermal unfolding of the protein was investigated using circular dichroism as a comparative technique. The data highlighted that the complex of the protein with ATP and Mg^2+^ enhanced the protein’s thermal stability. This enhancement was evident from the observed 1.17 °C increase in the *T*_m_ (Table 5). Ionic interactions, hydrogen bonds, hydrophobic interactions, and salt bridges improve the thermal stability of the protein. They increase the rigidity of protein which assists in the specificity of ligand binding to protein [49, 50]. This suggests that when Mg^2+^ is present, *Kp*PrAT is rigid to a state where it allows for an increase in thermal stability, this is seen in the increase in *T*_m_. From the far-UV CD, tertiary, and thermal stability studies, it can be proposed that the presence of the transitional metal ion is requisite for stable nucleotide binding.

The allure of ligand and protein binding specificity has captivated the scientific community. Isothermal titration calorimetry (ITC) enables the characterisation of protein-ligand binding without the use of a labelling molecule, like a fluorescent tag [51]. This technique quantifies the heat change resulting from the binding of a ligand to a protein, maintaining constant temperature, and typically reported as power (µJ·s^− 1^) [52]. The reaction can either be exothermic, releasing heat (∆*H*° < 0), or endothermic, absorbing heat (∆*H*° > 0). ITC offers several advantages, as it allows for the monitoring of interactions in non-immobilised conditions and provides valuable information regarding binding affinity and thermodynamic parameters, even in the events where the structural information of the protein is unavailable [53]. The isotherm gives insight into the enthalpy, binding affinity, stoichiometry, and Gibbs free energy of the reaction. The data was fitted using the independent model, which assumes that the ligand binds to a singular site on the protein. The presence of ATP resulted in exothermic reactions, indicating favourable enthalpy changes. This signifies that in both reactions, heat is released due to the association of the ligand with the protein. A favourable change in binding enthalpy is influenced by the formation of non-covalent interactions between the ligand and the protein, as well as the rearrangement of water molecules during the binding process [54]. However, the absence of the divalent metal ion is favourable in terms of entropy changes. The binding entropy is influenced by the solvation and conformational entropies. The solvation entropy typically favours the binding process because of the desolvation of the binding site when the ligand binds, whereas the conformational entropy tends to be unfavourable because both the protein and the ligand lose their conformational degrees of freedom upon binding [55]. Based on the ANS results and molecular dynamics (MD) simulations, the inclusion of Mg^2+^ in the solution led to the formation of a hydrophobic interactions within the ATP binding site. This hydrophobic environment influenced the binding entropy, primarily through changes in solvation entropy, as water was expelled from the site. However, despite this, the decrease in the rotational and translational degrees of freedom of protein-ligand binding ultimately led to an unfavourable overall entropy change [56] for the protein-ligand complex with the divalent metal ion. The phenomenon of enthalpy-entropy compensation provides the most suitable explanation for the favourable enthalpy and unfavourable entropy observed in the presence of Mg^2+^ in solution. It is theorised that a more negative binding enthalpy change would consequently lead to a negative binding entropy change, thereby promoting increased order within the system [57]. Additionally, both reactions are spontaneous, as indicated by the negative change in Gibbs free energy. The c-value, also known as the Wiseman value, determines the shape of the isotherm. A c-value falling within the range of 20–100 typically yields a good-fitted sigmoidal curve, indicating strong binding. Conversely, a value exceeding 1000 suggests moderate binding, enabling estimation of *∆H* and n. Moreover, c-values below 5 indicate a poor fit of the curve, making it challenging to estimate the thermodynamic parameters from the isotherm [58]. The Wiseman value can be calculated using the equation below:

$$c=\frac{n\left[P\right]}{{K}_{d}}$$

where *n* is the number of binding sites of the protein and [*P*] is the protein concentration in the sample cell. The c-value for *Kp*PrAT in the absence of Mg^2+^ exceeds 5, while for the protein in the presence of the divalent metal ion, it is slightly less than 5. This suggests that the estimated enthalpy and stoichiometry (*n*) for the protein in the absence of the metal ion are reliable. However, for the presence of the metal ion, these values may not be as reliable. Nonetheless, these estimations still provide valuable insights into the binding of the protein and ATP in the presence of the metal ion. The dissociation constant for *Kp*PrAT in the absence of a metal ion is 1.09 µM higher than for the protein in the absence of the divalent metal ion. The dissociation constant of *Kp*PrAT bound to ATP, without Mg^2+^ is comparable to an ATP-binding pseudokinase integrin-like kinase [59]. Furthermore, the binding affinity is inversely related to the dissociation constant. This means that the binding of ATP to *Kp*PrAT is stronger when Mg^2+^ is present in the solution. However, for both reactions the dissociation constant shows moderate binding. This is beneficial because it permits the association and dissociation of potential drug molecules, enabling them to compete with the natural substrate for the same binding site. Consequently, it serves as a viable drug target in drug design.

Comparing the same protein with different conformations can be represented by the C_α_ RMSD, which gives insight into the similarity between the two conformations. An assigned cut-off value of 3 Å has been proposed. This means that the smaller the deviation or values less than the aforementioned number indicate that the conformations are similar [60]. The presence of ATP and MgCl_2_ has the highest deviation (Figure 9A) compared to the other systems. This indicates that this system is divergent in conformation compared to the other systems, while the presence of only MgCl_2_ would suggest that it is the most comparable in conformation. Additionally, the divergence of the C_α_ RMSD gives information on whether the simulation is equilibrated [61]. A highly divergent system would indicate an unequilibrated system. The foundation of the statistical mechanics employed by algorithms in the analysis of molecular dynamic simulations data assumes thermodynamic equilibrium [62]. However, the primary structure of the protein is from PDB, which uses empirical approaches such as X-ray diffraction to obtain the 3D structure, and it is not the equilibrium protein structure [63]. Hence, the simulation should be equilibrated before the gathering of simulation trajectories. This phase in MD simulations ensures that the system is under optimal conditions. All the systems were equilibrated in the 250 ns simulations (Table 7). Furthermore, there was a divergence in all the systems between 0 and 100 ns, this is from the increase in the C_α_ RMSD at that time interval. However, they stabilise after 100 ns, additionally indicating that the systems were equilibrated. The C_α_ RMSF reflects the flexibility of a residue. The region 120–159 encompass the αC-helix, in the N-lobe. The αC-helix is considered to be dynamic and a regulatory component, as it binds to other parts of the protein kinase [26]. Residues 221–292 contain the changed Y/HRD and DFG motifs, which contain the catalytic and activation loops. Furthermore, the former motif is believed to be involved in forming and stabilising the active site of the protein [64]. The radius of gyration of protein in the presence of only ATP increases at ∼ 85 ns in the simulation and ranges between 24 and 29 Å, while the protein in complex with ATP and MgCl_2_ shows an increase in the (R_g_) at ∼ 180 ns and fluctuates between 24 and 29 Å. This means that the presence of ATP makes the protein more extended in the later stage of the simulation, while the absence of the nucleotide has no notable change in the R_g_. The 2D interaction plots show that the presence of Mg^2+^ leads to the expulsion of water molecules around the nitrogenous base of the ATP, and this, coupled with increased non-polar amino acids, creates a hydrophobic region. Furthermore, more water molecules surround the phosphate group of ATP, when the divalent metal ion is present. When the 2D interaction plots are complemented with the interaction fraction plots, a notable increase in H-bonds is observed when the protein is complexed with ATP and Mg^2+^. The interaction fraction plot shows the increase in ionic interactions without the divalent cation. From the 2D interaction plots it can also be suggested that Arg 113 and 159 stabilise the γ-phosphate in ATP in both the absence or presence of Mg^2+^ and Arg 238 as well as Gly 265 stabilise the ATP at the nitrogenous base. The absence of the aforementioned amino acids when Mg^2+^ may lead to the destabilisation of the adenine moiety, which may be more dynamic. The stabilisation and decreased polarity in the adenosine moiety is essential to allow a conformation suitable for substrate binding. The ANS binding studies complement the 2D interaction plots and interaction fraction plots. The results propose that the presence of Mg^2+^ in the *Kp*PrAT: ATP complex increases the hydrophobicity in the ATP binding site, allowing for substrate binding.

The criteria for identifying a plausible druggable site include a substantial volume and a potentially hydrophobic constitution [36]. SiteMap analysis indicates that Site 1 possesses the characteristics of a ligand-binding and druggable site. This finding is supported by a SiteScore > 0.80 and a D-Score > 0.83 across all systems. Furthermore, the D-Score provides insights into the binding site’s hydrophobicity. The binding of the divalent metal ion to the protein not only increased the D-Score but also expanded the volume of the binding sites. This implies that the metal ion induces a conformation that is hydrophobic and conducive to substrate binding. These findings find support in ANS studies and 2D interaction plots. This discovery holds significant importance for protein crystallisation. It suggests that the inclusion of Mg ions in the crystallisation process is crucial, as they contribute to stabilising the protein’s dynamics, ultimately enhancing the molecule’s ability to crystallise.

## Conclusion

The overexpression, purification, and structural characterisation of *Kp*PrAT were successful. Far-UV CD spectra presented that even with Mg^2+^, the spectra were representative of a predominantly α-helical protein. Dichroweb results showed an increase in β-strands, which are key in substrate binding. Extrinsic fluorescent spectroscopy, ANS, and mant-ATP were used as fluorescent probes, highlighting the presence of a hydrophobic pocket. This pocket is potentially an ATP binding site from the mant-ATP studies. Isothermal titration calorimetry played a crucial role in elucidating the binding of ATP, both with and without Mg^2+^. This technique revealed a higher affinity for ATP binding to *Kp*PrAT when the divalent metal ion was present in solution. In summary, the binding of ATP to the protein is thermodynamically favourable in terms of enthalpy and occurs spontaneously. Furthermore, the presence of Mg^2+^ stabilises this pocket for ATP binding, which was observed from ANS, MD simulations, and the thermal stability studies. This study was effective in emphasising that the presence of Mg^2+^ induces a conformation in *Kp*PrAT that favours nucleotide binding. A crystal structure of *Kp*PrAT: Mg^2+^ and either with or without ATP could give further insight into the binding site and potential catalytic mechanism of the enzyme. The presence of the divalent cation decreases the dynamism of *Kp*PrAT, which is favourable for protein crystal growth. Future work should focus on exploring Grx as a potential substrate of *Kp*PrAT to obtain the function of the enzyme and X-ray crystallography. Furthermore, it is crucial to consider the optimal conformational state influenced by the presence of Mg^2+^ when developing the workflow for high-throughput virtual screening (HTVS). This is significant because the model system’s capability is limited to predicting the most probable compounds that will interact within the cavity, as indicated by the D-score metrics. Therefore, it is of utmost importance to use the best conformational state in HTVS and validate the docking through empirical methods. The data obtained from this research contributes to bringing novel, prospective therapeutic agents against ESKAPE bacteria, especially in Africa.

### Electronic Supplementary Material

Below is the link to the electronic supplementary material.

Supplementary Material 1

## Data Availability

No datasets were generated or analysed during the current study.
