# Supplementary material for: Expression, Purification and Biophysical Characterisation of Klebsiella Pneumoniae Protein Adenylyltransferase: A Systematic Integration of Empirical and Computational Modelling Approaches
Source: Protein J. 2024 Jul 9;43(4):751–70. doi: 10.1007/s10930-024-10210-3 (PMC11345332; doi:10.1007/s10930-024-10210-3)
Supplement: Supplementary file 1 — Supplementary Material 1 [file 10930_2024_10210_MOESM1_ESM.pdf]

Expression, purification and biophysical characterisation of *Klebsiella pneumoniae* protein adenylyltransferase: A systematic integration of empirical and computational modelling approaches

Reabetswe Maake[a], and Ikechukwu Achilonu[a]*

[a] Protein Structure-Function and Research Unit, School of Molecular and Cell Biology, Faculty of Science, University of the Witwatersrand, Braamfontein, Johannesburg 2050, South Africa

Supplementary data

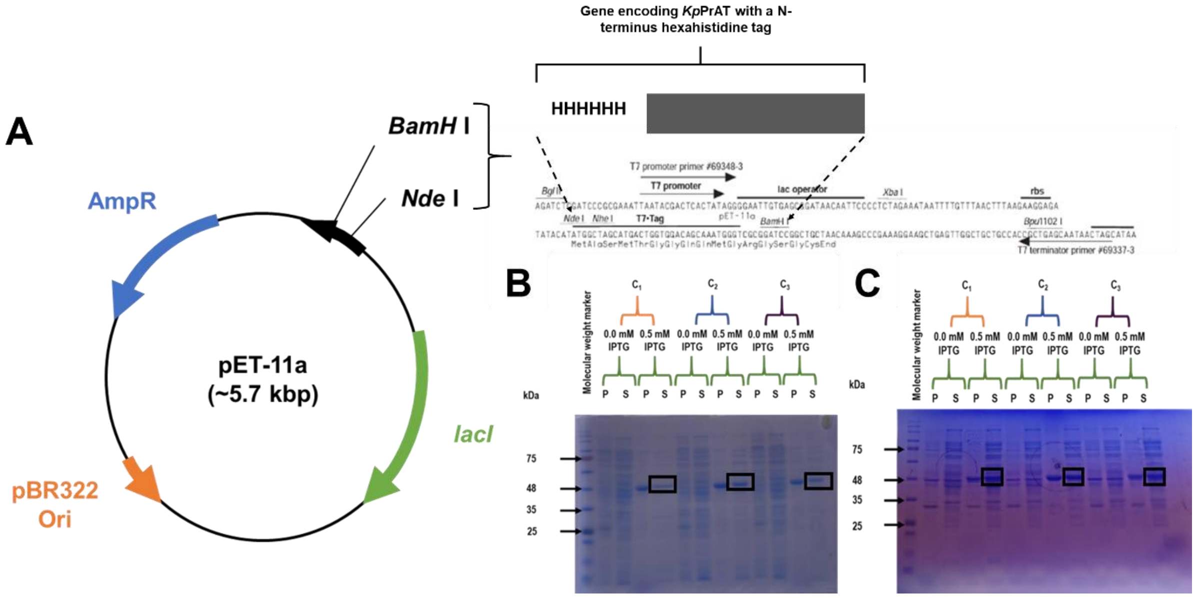

Figure S1. Schematic of the pET-11a-*Kp*PrAT vector construct and 12% (*w/v*) SDS-PAGE gels for the analysis of expression trials of *Kp*PrAT. (A) Illustrates the vector construct of *Kp*PrAT, cloned into a pET-11 expression system. The T7 *E. coli* cells were transformed with the *Kp*PrAT-pET-11a vector construct. IPTG concentration was varied, 0 or 0.5 mM. (B) The expression trials were conducted at 30°C, for 6 hrs. The protein was soluble in the supernatant at 0.5 mM IPTG, encapsulated in a black box. (C) Trials were carried out at 15°C, for 24 hrs. Protein solubility, in the supernatant, increased.

```
CLUSTAL O(1.2.4) multiple sequence alignment


sp|B5XQE2|SELO_KLEP3      MTLSFTTHWRDELPDFYTSLLPTPLDNARLIWRNAPLAQQLGVPDALFAPENGAGVWGGE  60
pdb|6K20|A                MTLSFITRWRDELPATYTTLSPTPLNNARLIWHNAELANTLGIPSSLFK--NGAGVWGGE  58
                          ***** *:******  **:* ****:******:** **: **:*.:**   *********

sp|B5XQE2|SELO_KLEP3      ALLPGMSPLAQVYSGHQFGAWAGQLGDGRGILLGEQQLADGRRYDWHLKGAGLTPYSRMG  120
pdb|6K20|A                TLLPGMSPLAQVYSGHQFGVWAGQLGDGRGILLGEQRLADGTTMDWHLKGAGLTPYSRMG  118
                          :******************.****************:****   ****************

sp|B5XQE2|SELO_KLEP3      DGRAVLRSTIRESLASEAMHALGIPTTRALAMVTSDTPIYRERVEPGAMLMRVAESHVRF  180
pdb|6K20|A                DGRAVLRSTIRESLASEAMHYLGIPTTRALSIVTSDSPVYRETVEPGAMLMRVAPSHLRF  178
                          ******************** *********::****:*:*** *********** **:**

sp|B5XQE2|SELO_KLEP3      GHFEHFYYRREPQKVQQLADYVIRHHWPQLQDEADKYLLWFRDVVTRTAQTIASWQTVGF  240
pdb|6K20|A                GHFEHFYYRREPEKVRQLADFAIRHYWSHLADDEDKYRLWFTDVVARTASLIAQWQTVGF  238
                          ************:**:****:.***:* :* *: *** *** ***:***. **.******

sp|B5XQE2|SELO_KLEP3      AHGVMNTDNMSILGLTIDYGPYGFLDDFQPDFICNHSDYQGRYSFENQPAVGLWNLQRLA  300
pdb|6K20|A                AHGVMNTDNMSLLGLTLDYGPFGFLDDYEPGFISNHSDHQGRYSFDNQPAVALWNLQRLA  298
                          ***********:****:****:*****::*.**.****:******:*****.********

sp|B5XQE2|SELO_KLEP3      QSLSPFISAEALNAALDEYQHALLTAYGQRMRDKLGLFSQQKGDNDLLDGLFALMIREKS  360
pdb|6K20|A                QTLSPFVAVDALNEALDSYQQVLLTHYGQRMRQKLGFMTEQKEDNALLNELFSLMARERS  358
                          *:****::.:*** ***.**:.*** ******:***::::** ** **: **:** **:*

sp|B5XQE2|SELO_KLEP3      DYTRTFRLLSHSEQLSAASPLRDEFIDRAAFDSWFAGYRARLRDEQVDDAQRQQRMQGVN  420
pdb|6K20|A                DYTRTFRMLSLTEQHSAASPLRDEFIDRAAFDDWFARYRGRLQQDEVSDSERQQLMQSVN  418
                          *******:** :** *****************.*** **.**::::*.*::*** **.**

sp|B5XQE2|SELO_KLEP3      PALVLRNWLAQRAIEQAEAGDMGELERLHAALADPFTDREDDYVRRPPDWGKRLEVSCSS  480
pdb|6K20|A                PALVLRNWLAQRAIEAAEKGDMTELHRLHEALRNPFSDRDDDYVSRPPDWGKRLEVSCSS  478
                          *************** ** *** **.*** ** :**:**:**** ***************
```

Figure S2. Sequence alignment of *Ec*PrAT and *Kp*PrAT. The Clustal Omega tool was used to align the sequences of *E. coli* SelO protein (PDB:6K20) and the *K. pneumoniae* SelO protein, *Kp*PrAT. There is an estimated 78% sequence alignment between the two protein sequences.

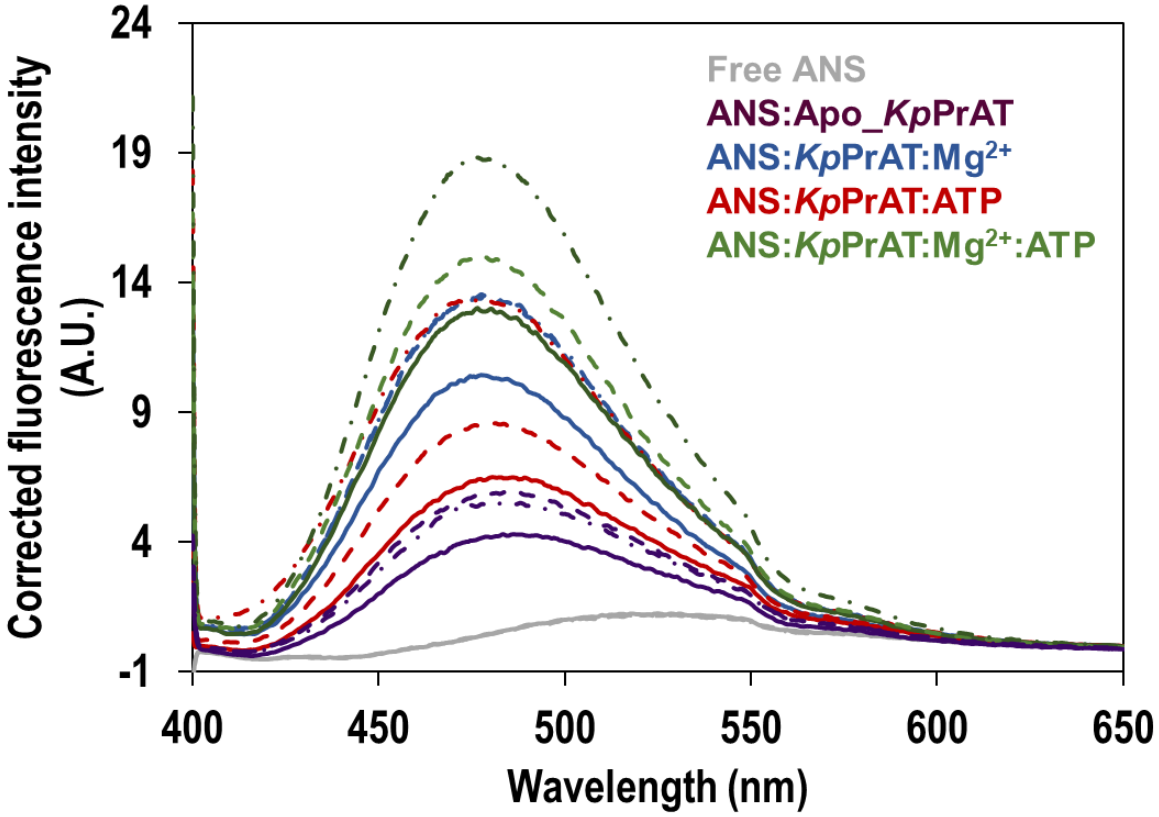

Figure S3. Fluorescence emission spectra of ANS associating with *Kp*PrAT, either with or without ATP and/or Mg^{2+}. The image illustrates the replicates for each condition, free ANS, ANS:*Kp*PrAT, ANS:*Kp*PrAT:Mg^{2+}, and ANS:*Kp*PrAT:Mg^{2+}:ATP.

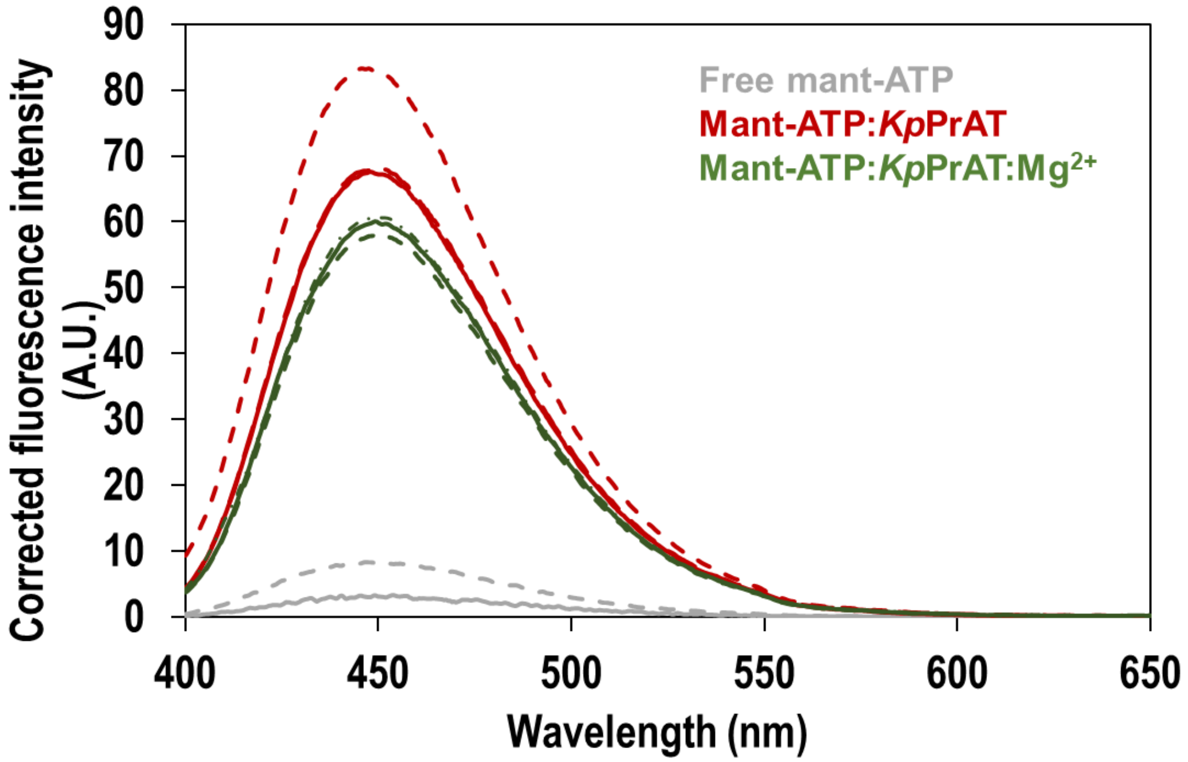

Figure S4. Fluorescence emission spectra of (A) mant-ATP. The image illustrates the replicates of each nucleotide binding to *Kp*PrAT, either with or without Mg^{2+}.

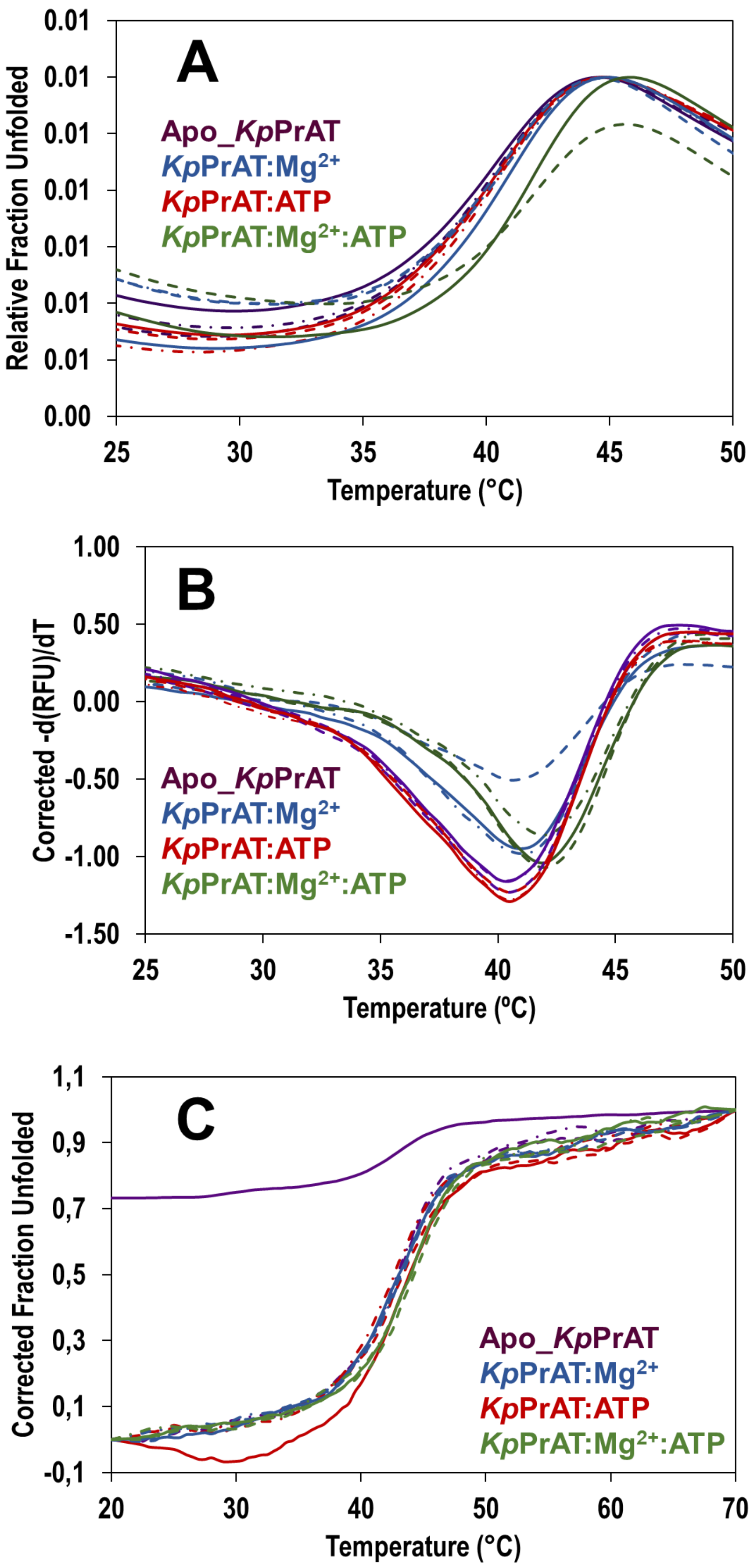

Figure S5. Thermal unfolding curves. SYPRO-Orange is represented as (A) fluorescence emission spectra in fraction unfolded and (B) melting curves. (C) Thermal unfolding curves with circular dichroism. The image displays the replicates for each condition, Apo_*Kp*PrAT, *Kp*PrAT:ATP, *Kp*PrAT:Mg^{2+}, and *Kp*PrAT:Mg^{2+}:ATP.

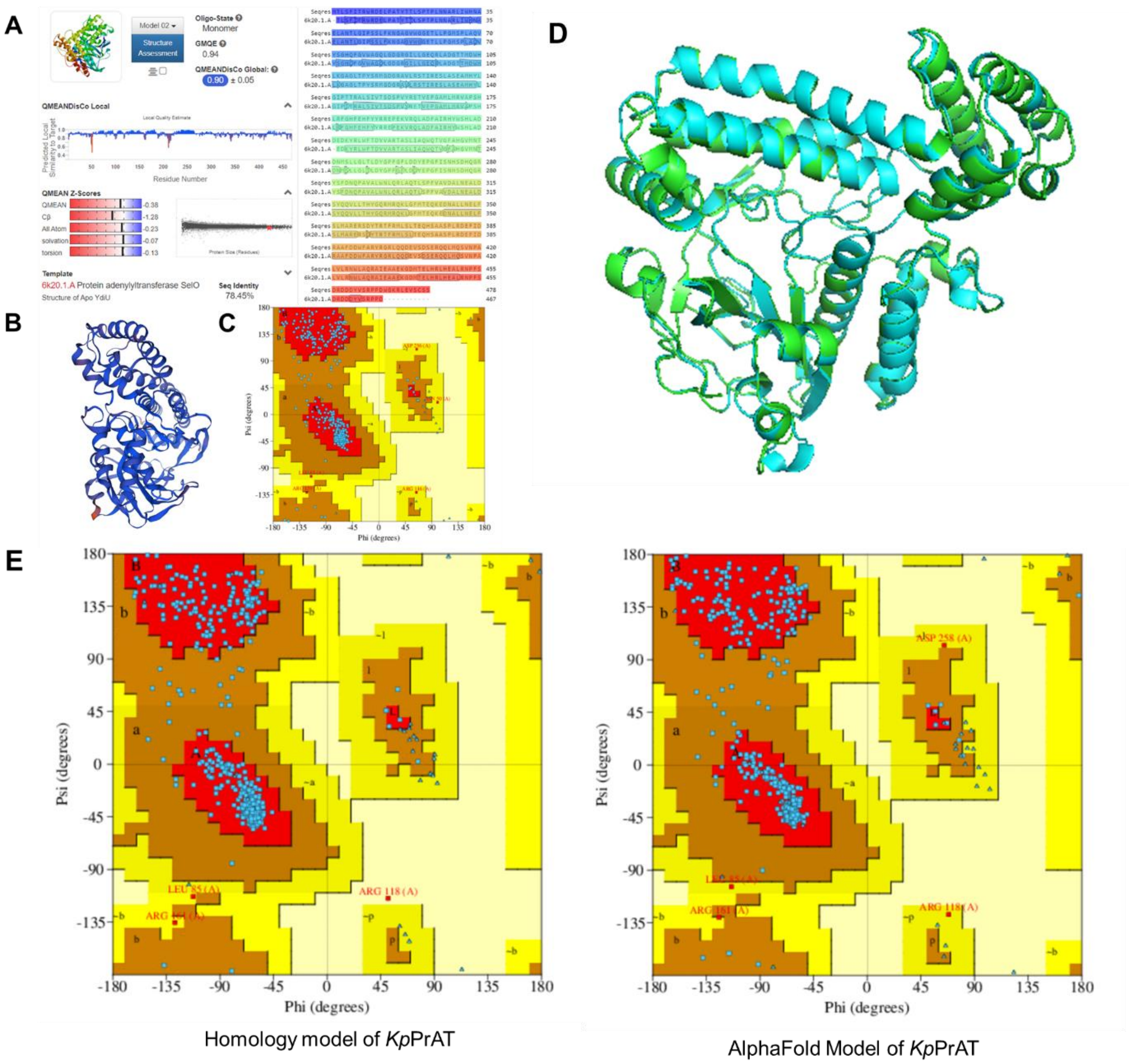

Figure S6. Validation of the *Kp*PrAT model with the use of the (A) Swiss model tool, which shows a 78.45% sequence identity between *Kp*PrAT and the template it was built on, *E. coli* adenylyltransferase. (B) The homology model of *Kp*PrAT built by Swiss model, with a root-mean-squared deviation (RMSD) of 0.062 and 465 residues that align when the model was superimposed with the template using PyMol. (C) Ramachandran plot represents the stereochemistry and geometry of the side chains. There were 95.92% of residues in the allowed region, while there were no residues in the disallowed regions. (D) Homology model of *Kp*PrAT (turquoise) aligned with the AlphaFold model (green), generated using PyMol. (E) The Ramachandran plots show that both models have at least 90% of residues in the allowed region.

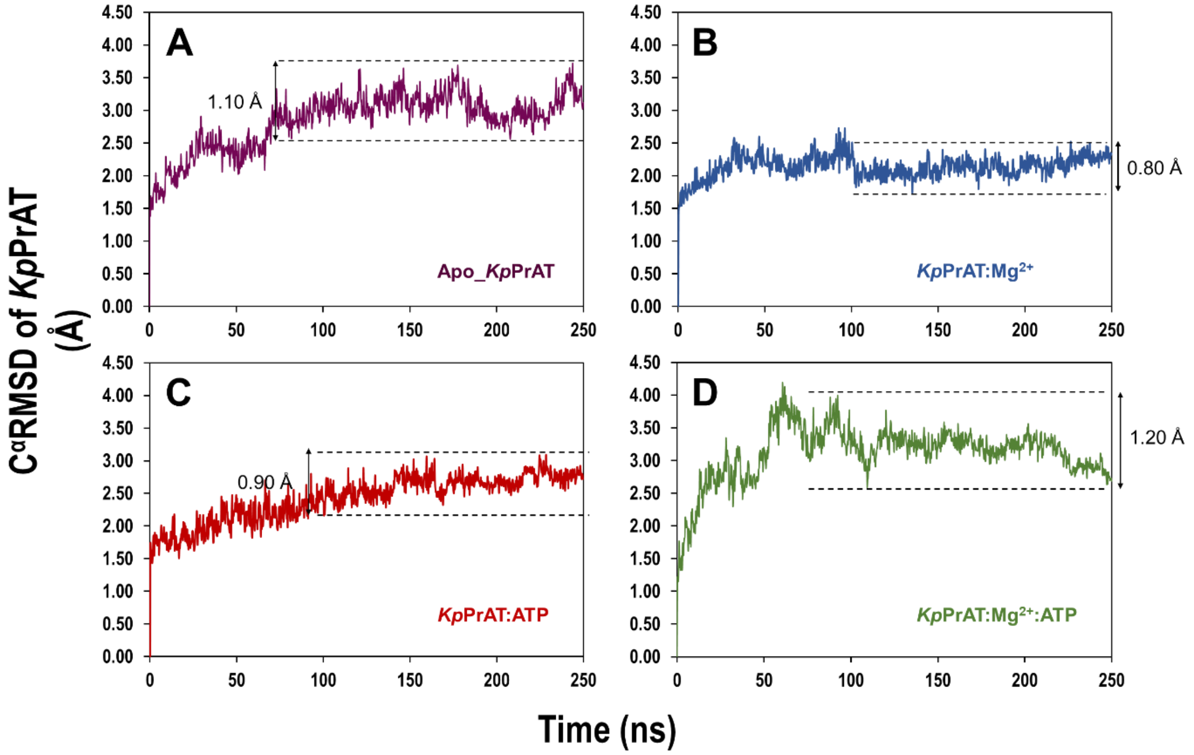

Figure S7. The root-mean-square deviation (RMSD) of the Cα atoms of *Kp*PrAT over a 250 ns simulation time. (A) Apo_*Kp*PrAT, (B) *Kp*PrAT:Mg^{2+}, (C) *Kp*PrAT:ATP, and (D) *Kp*PrAT:Mg^{2+}:ATP.

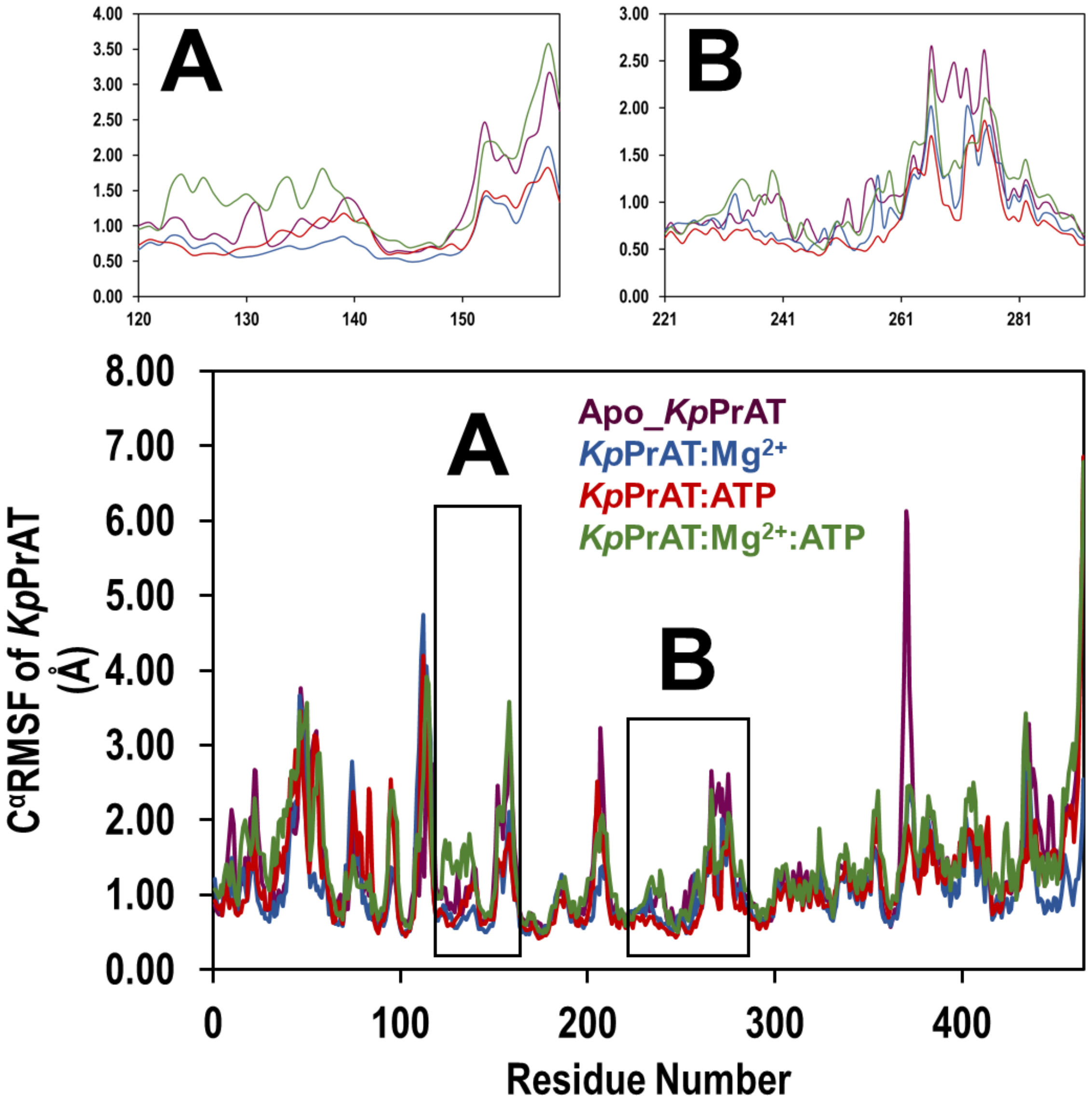

Figure S8. The root-mean-square fluctuation (RMSF) of the Cα atoms of *Kp*PrAT over a 250 ns simulation time. The zoomed in region of the residue number between (A) 120-159, and (B) 221-292.

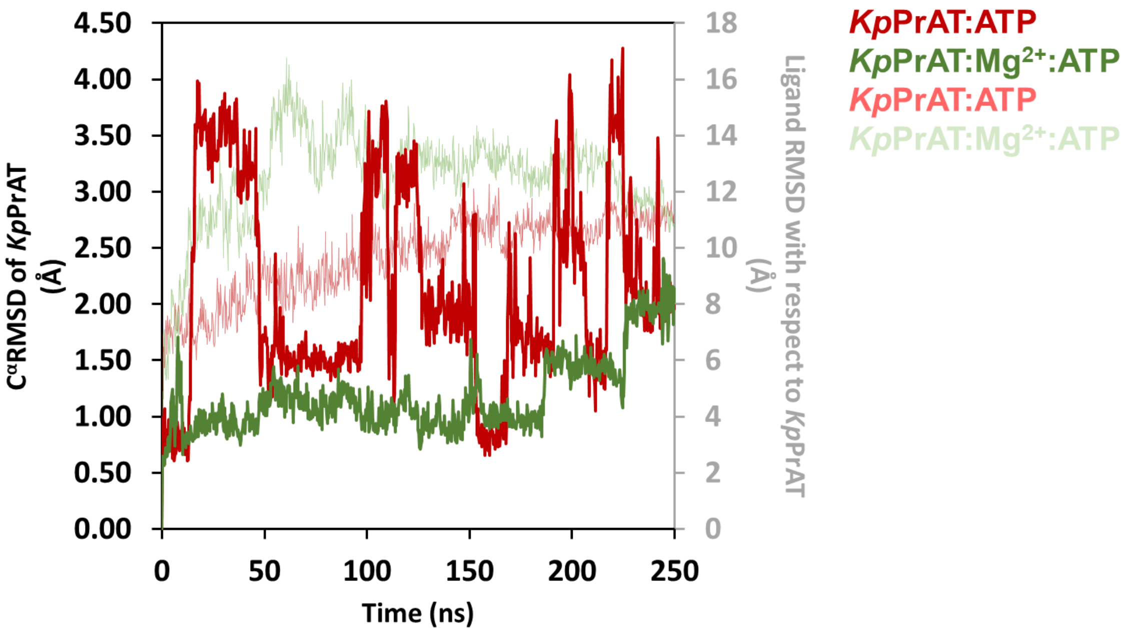

Figure S9. The root-mean-square deviation (RMSD) of the Cα atoms of *Kp*PrAT in complex with ATP, either in the absence or presence of $MgCl_2$ salt, over a 250 ns simulation time. The secondary y-axis is the ligand RMSD with respect to *Kp*PrAT in complex with ATP either in the absence or presence of $MgCl_2$ salt.

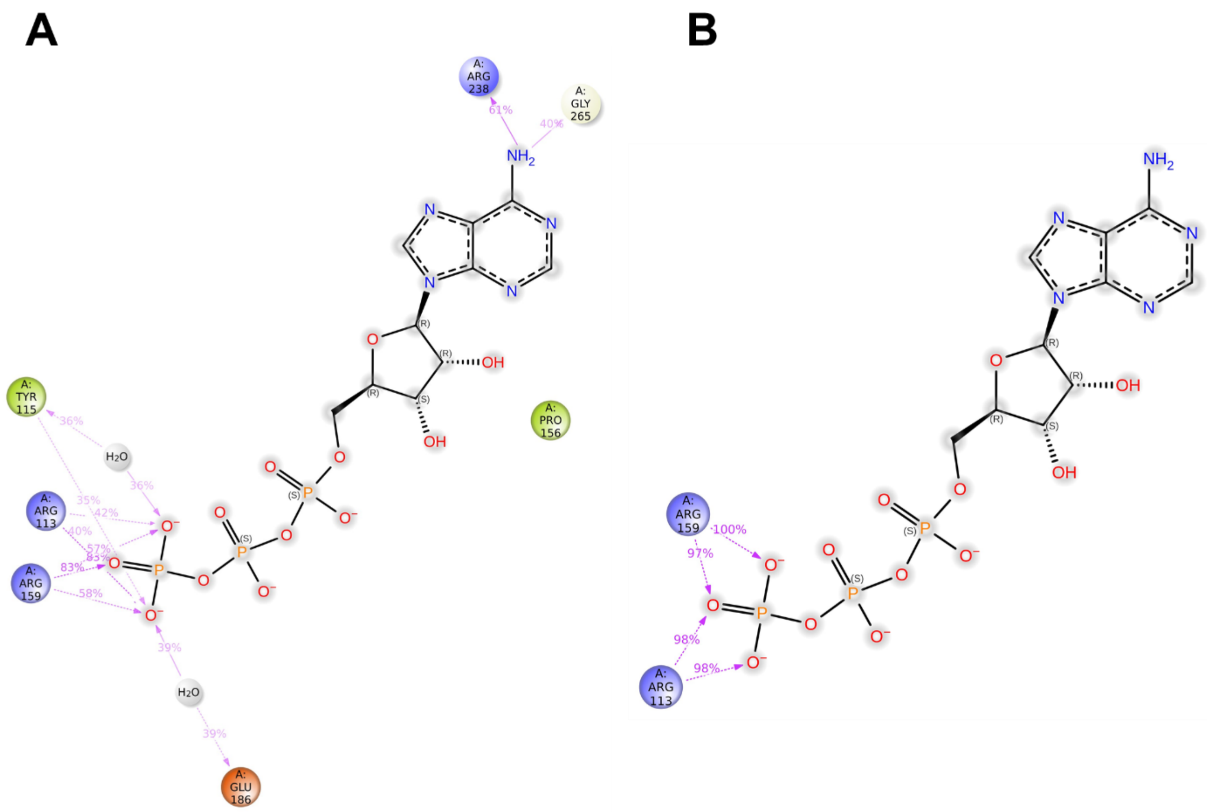

Figure S10. 2D interaction plot showing the association of *Kp*PrAT with ATP, across the 250 ns simulation. This interaction was monitored for condition of protein and ATP either in the (A) presence of $MgCl_2$ and (B) absence of $MgCl_2$.
